# The inhibition of PINK1/Drp1-mediated mitophagy by hyperglycemia leads to impaired osteoblastogenesis in diabetes

**DOI:** 10.1016/j.isci.2024.111519

**Published:** 2024-12-03

**Authors:** Xiao-jing Chen, Yu-ying Yang, Zheng-can Pan, Jing-zun Xu, Tao Jiang, Lin-lin Zhang, Ke-cheng Zhu, Deng Zhang, Jia-xi Song, Chun-xiang Sheng, Li-hao Sun, Bei Tao, Jian-min Liu, Hong-yan Zhao

**Affiliations:** 1 Department of Endocrine and Metabolic Diseases, Shanghai Institute of Endocrine and Metabolic Diseases, Ruijin Hospital, Shanghai Jiao Tong University School of Medicine, Shanghai, China; 2 Shanghai National Clinical Research Center for Metabolic Diseases, Key Laboratory for Endocrine and Metabolic Diseases of the National Health Commission of the PR China, Shanghai Key Laboratory for Endocrine Tumor, State Key Laboratory of Medical Genomics, Ruijin Hospital, Shanghai Jiao Tong University School of Medicine, Shanghai, China; 3 Department of Endocrinology, Xinhua Hospital, Shanghai Jiao Tong University School of Medicine, Shanghai, China

**Keywords:** Physiology, Molecular biology, Cell biology

## Abstract

Impaired bone quality and increased fracture risk are cardinal features of the skeleton in diabetes mellitus. Hyperglycemia-induced oxidative stress is proposed as a potential underlying mechanism, but the precise pathogenic mechanism remains incompletely understood. In this investigation, osteoblasts under high glucose exhibited heightened levels of reactive oxygen species, impaired mitochondrial membrane potential, and profound inhibition of late-stage osteoblast differentiation. Further analyses uncovered that high glucose resulted in the downregulation of the PINK1/Drp1 pathway in osteoblasts, consequently leading to impaired mitophagy. Conversely, the upregulation of PINK1/Drp1 pathway activated mitophagy, which restored the differentiation capacity of osteoblasts. Notably, in an STZ-induced diabetic mouse model, BMP9 upregulated the expression of PINK1/Drp1 in the bone tissue, leading to an improvement in bone quality and bone mineral density. These findings suggest that the PINK1/Drp1 pathway might be a potential therapeutic target to enhance osteogenic differentiation and treat diabetic osteoporosis.

## Highlights

• PINK1/Drp1-mediated mitophagy contributes to osteoblastogenesis
• Hyperglycemia suppresses PINK1/Drp1-mediated mitophagy, causing impaired osteoblastogenesis
• BMP9 reverses hyperglycemia-induced impaired osteoblastogenesis by activating mitophagy
• BMP9 treats diabetic osteoporosis in mice by activating PINK1/DRP1-mediated mitophagy

## Graphical abstract

**Figure:**
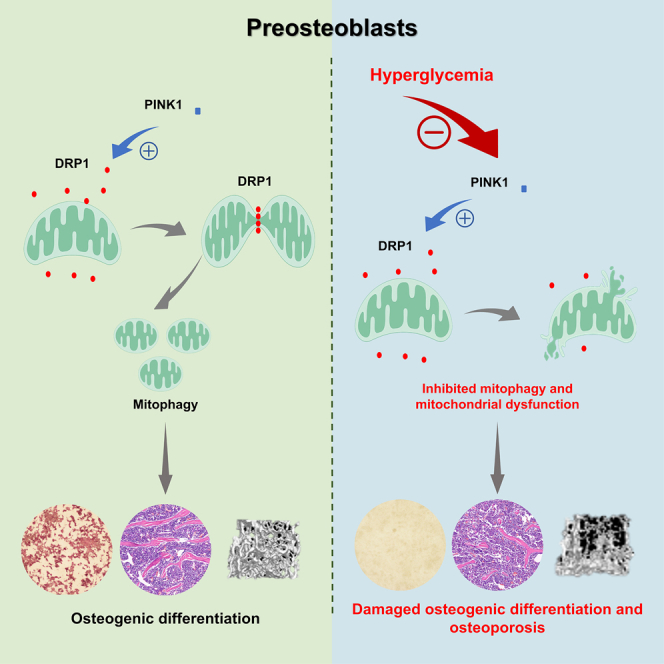

## Introduction

As with many other diabetic complications, the poor bone quality and increased fracture risk in diabetes has already aroused great concerns⁠.^1^^,^^2^ It was well recognized that hyperglycemia exposure inhibits proliferation and differentiation of osteoblasts,^3^ but its specific mechanism is still not clear, and no definitive pharmaceutical remedies for diabetic osteoporosis are available except for those traditional medications to treat primary osteoporosis.

Energy metabolism is an important physiological basis for maintaining cell function. The function of mitochondria, as the hub of energy metabolism, is the key to maintaining normal life activities. Mitochondrial dysfunction-related diseases have expanded from rare monogenic disorders to common polygenic diseases, including metabolic, cardiovascular, neurodegenerative, and neuromuscular diseases in nowadays.^4^^,^^5^ Lots of effort was made to explore signaling pathways to reverse mitochondrial dysfunction.^6^ Recent studies have suggested a mutual cross-talk between bone remodeling and glucose homeostasis.^7^^,^^8^ So far, the best characterized metabolic pattern for osteoblast maturation is the glucose metabolism reprogramming from oxidative phosphorylation (OXPHOS) toward aerobic glycolysis, coupling with diminished mitochondrial respiration.^9^^,^^10^ Suppression of glucose metabolism restricts osteoblast differentiation.^11^ Thus, it is interesting to investigate whether and how the disturbed metabolic reprogramming causes impaired osteoblastogenic differentiation and poor bone quality in diabetes, and how to reverse it.

Mitophagy is a mechanism that eliminates damaged mitochondria and controls mitochondrial quality and quantity. Simultaneously, mitophagy mediates metabolic reprogramming, either into an OXPHOS phenotype or into a glycolytic phenotype, to satisfy the metabolic requirements of cells.^12^^,^^13^ Mitophagy dictates stem cell fate by facilitating glycolytic phenotype swift.^14^ The deficiency of mitophagy induces reactive oxygen species (ROS) stress, and leads to the development of neurodegenerative disorder,^15^ cardiomyopathy,^16^ and acute kidney injury.^17^ In type 2 diabetes patients, mitophagy pathway gene expression was downregulated.^18^ In β-cells, decreased glucose-responsive insulin secretion was associated with impaired mitophagy.^19^ Mitophagy is mediated by PTEN-induced kinase 1 (PINK1)/Parkin pathway. PINK1 is a mitochondrial serine/threonine-protein kinase. It can sense mitochondrial stress, accumulate on defective mitochondria, recruit Parkin, and finally trigger mitophagy.^20^ Loss of PINK1/Parkin may contribute to several diseases, such as Parkinson’s disease.^21^ As to bone metabolism, PINK1-mediated mitophagy is involved in cartilage degeneration seen in osteoarthritis.^22^^,^^23^^,^^24^

Therefore, our research interest is what role mitophagy plays in hyperglycemia-induced osteoblastogenic defects and whether the intervention of related mechanisms can restore diabetic osteoporosis.

## Results

### High glucose level inhibited osteogenic differentiation and respiration rate of MC3T3-E1 cells

**Figure 1:**
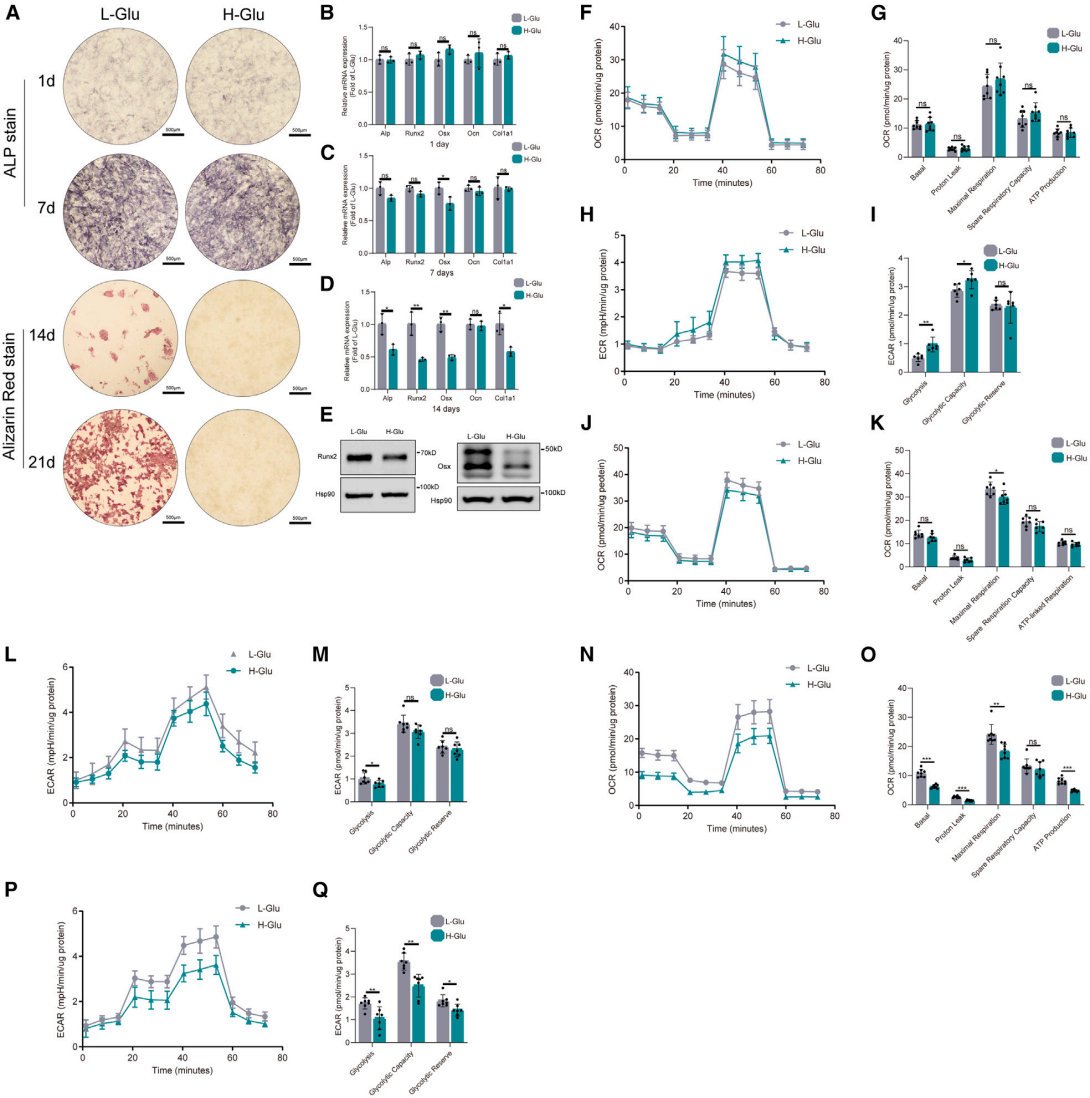
High glucose inhibited osteoblastogenic differentiation and respiration rate of MC3T3-E1 cells The concentration of glucose in the medium of the L-Glu group was 5.5 mmol/L, and that in the medium of the H-Glu group was 25 mmol/L. (A) ALP staining was performed in L-Glu and H-Glu cultured MC3T3-E1 cells after 1 and 7 days of osteogenic induction. Alizarin red S staining was performed in L-Glu and H-Glu cultured MC3T3-E1 cells after 14 and 21 days of osteogenic induction. Scale bars, 500 μm. (B–D) MC3T3-E1 cells were cultured in an osteogenic medium for 1, 7, and 14 days. Expressions of osteogenic markers at different time points were determined by RT-qPCR. (E) MC3T3-E1 cells were cultured in osteogenic medium for 14 days. Expressions of Runx2 and Osx were determined by WB. (F–Q) MC3T3-E1 cells were cultured in osteogenic medium for 1, 7, and 14 days. Mitochondrial oxidative capacity and glycolytic capacity were measured in real time. Basal respiration, ATP-linked respiration, maximal respiration, spare respiratory capacity, basal, proton leak, glycolysis, glycolytic capacity, and glycolytic reverse were calculated by WAVE software. Data presented as mean ± SD. *n* = 3 biological replicates. One-way ANOVA was used for comparison among multiple groups. ∗*p* < 0.05; ∗∗*p* < 0.01; ∗∗∗*p* < 0.001. Ns, no significance.

To investigate the effect of high glucose level on the osteoblastogenesis of MC3T3-E1 cells *in vitro*, alkaline phosphatase (ALP) and Alizarin red staining were performed. Alizarin red staining in H-Glu group showed significantly fewer mineralization nodules compared with L-Glu group in the late stage (14 days, 21 days) of osteogenic differentiation. However, there was no significant difference in ALP staining between the two groups in the early stage (1 day, 7 days) of osteogenic differentiation (Figure 1A). Furthermore, the mRNA expression levels of Runx2, Osx, Col1a1, and ALP were reduced in H-Glu group at day 14, while there was no significant difference between H-Glu and L-Glu group at day 1, and only Osx expression decreased significantly in the H-Glu group at day 7 (Figures 1B and 1D). Consistently, the western blot (WB) results showed that the protein levels of Runx2 and Osx were decreased in H-Glu group at day 14 (Figure 1E). These results suggest that high glucose inhibits the late stage of osteogenic differentiation in MC3T3-E1 cells.

Since the disruption of energy metabolism in osteoblasts may lead to impaired osteoblast function,^25^^,^^26^ we then detected two key energy metabolic pathways, aerobic respiration and glycolysis in MC3T3-E1 cells using the Seahorse extracellular flux analyzer. On day 1 of osteogenic differentiation, no noteworthy distinctions of basal respiration, maximal respiration, spare respiration capacity, and ATP-linked respiration, which are pivotal parameters that underscore mitochondrial functionality, were observed between H-Glu group and L-Glu group (Figures 1F and 1G). As to cellular glycolytic function, H-Glu group exhibited elevated glycolysis and glycolytic capacity compared to the L-Glu group, with no statistically significant variance in glycolytic reversal between the two groups (Figures 1H and 1I). Subsequent to 7 days of osteogenic induction, exclusive reductions in maximal respiration and glycolysis were discerned in H-Glu group relative to L-Glu group (Figures 1J–1M). Following a 14-day osteogenic differentiation period, the mitochondrial stress test revealed a substantial attenuation in basal, maximal, and ATP-linked respiration in H-Glu cells as contrasted with those in L-Glu group (Figures 1N and 1O). Simultaneously, outcomes from the glycolytic rate examination exhibited notable diminutions across glycolysis, glycolytic capacity, and glycolytic reversal in H-Glu cells in comparison to L-Glu counterparts (Figures 1P and 1Q). Collectively, these findings provide evidence that the late stage of osteogenic differentiation is notably affected by a discernible inhibition of osteoblastic mitochondrial function induced by the high glucose level.

### High glucose level did not affect mitochondrial biogenesis in MC3T3-E1 cells

**Figure 2:**
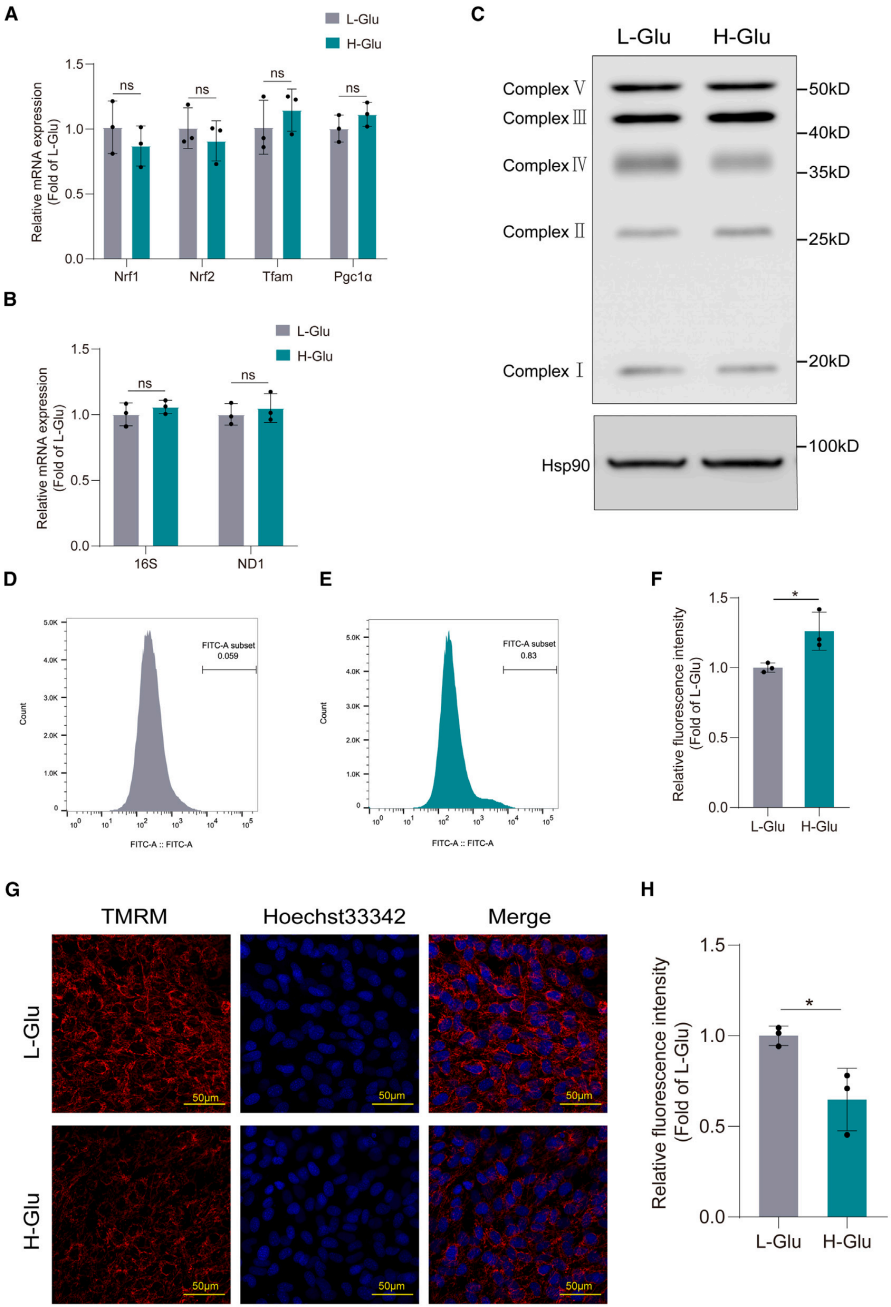
High glucose did not affect mitochondrial biogenesis but elevated ROS production and decreased mitochondrial membrane potential (MMP) in MC3T3-E1 cells (A) MC3T3-E1 cells were cultured in osteogenic medium for 14 days. The total DNA of the cells was extracted. Using HK2 as reference gene, expression ND1 and 16s were determined by RT-qPCR. (B) MC3T3-E1 cells were cultured in osteogenic medium for 14 days. Expressions of mitochondrial biogenesis markers were determined by RT-qPCR. (C) MC3T3-E1 cells were cultured in osteogenic medium for 14 days. Expressions of respiratory chain complexes were determined by WB. (D–F) MC3T3-E1 cells were cultured in osteogenic medium for 14 days. ROS generation was observed by using flow cytometry following staining with DCFH-DA. (G and H) MC3T3-E1 cells were cultured in osteogenic medium for 14 days. MMP of living cells was assessed by the Image-iT TMRM Reagent. Scale bars, 50 μm. Data presented as mean ± SD. *n* = 3 biological replicates. A t test was used for comparison between two groups. One-way ANOVA was used for comparison among multiple groups. ∗*p* < 0.05; ∗∗*p* < 0.01; ∗∗∗*p* < 0.001. Ns, no significance.

Mitochondrial proliferation may indicate the engagement of feedback loops to compensate for compromised mitochondrial function. Alterations in gene expression profile involved in mitochondrial biogenesis, and changes of concentrations and activities of candidate respiratory complexes in cells may lead to energetic dysfunction.^27^ Hence, the peroxisome proliferator-activated receptor gamma coactivator 1a (PGC-1α), nuclear respiratory factors (NRF-1 and NRF-2), and mitochondrial transcription factor A (TFAM) that are the key regulators of mitochondrial biogenesis were determined in osteoblasts. The results showed that there was no difference in the expression of the aforementioned genes between H-Glu and L-Glu groups (Figure 2A).

Mitochondrial biogenesis involves the integration of multiple transcriptional pathways controlling both nuclear and mitochondrial gene expression. For example, mitochondrial DNA (mtDNA) transcription is activated by the family of PGC-1 proteins (PGC-1α and PGC-1β), from which PGC-1α is considered the master regulator of mitochondrial biogenesis. The pathway is initiated by PGC-1α activation, followed by stimulation of a series of nuclear transcription factors, that is the NRF-1 and NRF-2 and by the increase in expression of TFAM, the final effector of mtDNA transcription and replication.^28^

To further measure mitochondrial biogenesis, we quantitated relative mtDNA copy number by qPCR in MC3T3-E1 cells. As shown in Figure 2B, mtDNA copy number in osteoblasts of H-Glu group remained similar to L-Glu group. Additionally, we examined the relative levels of the 5 OXPHOS complexes in mitochondria by WB. The result was in accordance with the mitochondrial biogenesis gene expression. Mitochondrial respiratory chain complex protein levels were not significantly different between the two groups (Figure 2C). These results demonstrate that high glucose level does not affect the mitochondrial biogenesis of MC3T3-E1 cells and suggest that high glucose level may affect mitochondrial function through other mechanisms.

### High glucose level elevated ROS production and decreased MMP in MC3T3-E1 cells

Mitochondria is one of the main sources of ROS, as it utilizes oxygen for energy production. Mitochondrial dysfunction may lead to increased radical production and consequent “oxidative stress.” Therefore, cell ROS level is one of the recognized indicators to evaluate mitochondrial function.^29^ The flow cytometry results in this study showed that the number of positively stained cells in H-Glu group was significantly higher than that in L-Glu group (Figures 2D and 2E). Fluorescence intensity quantitative analysis revealed that the ROS level in H-Glu group was significantly higher than that in L-Glu group (Figure 2F).

Mitochondrial function can also be assessed through monitoring changes in MMP. A decrease in the MMP indicates mitochondrial dysfunction.^30^ We examined the MMP using a cell-permeant dye tetramethylrhodamine (TMRM), which can accumulate in active mitochondria with intact membrane potentials. The TMRM signals in living cells were observed by confocal laser microscopy, quantitative analysis of TMRM fluorescence intensity in images showed that the average fluorescence intensity of cells in H-Glu group was 36% lower than that in L-Glu group. The MMP of H-Glu group cells were reduced (Figures 2G and 2H). These results suggest that high glucose level impairs mitochondrial function in MC3T3-E1 cells.

### RNA-seq data analysis of differentially expressed genes in MC3T3-E1 cells cultured in L-Glu and H-Glu

**Figure 3:**
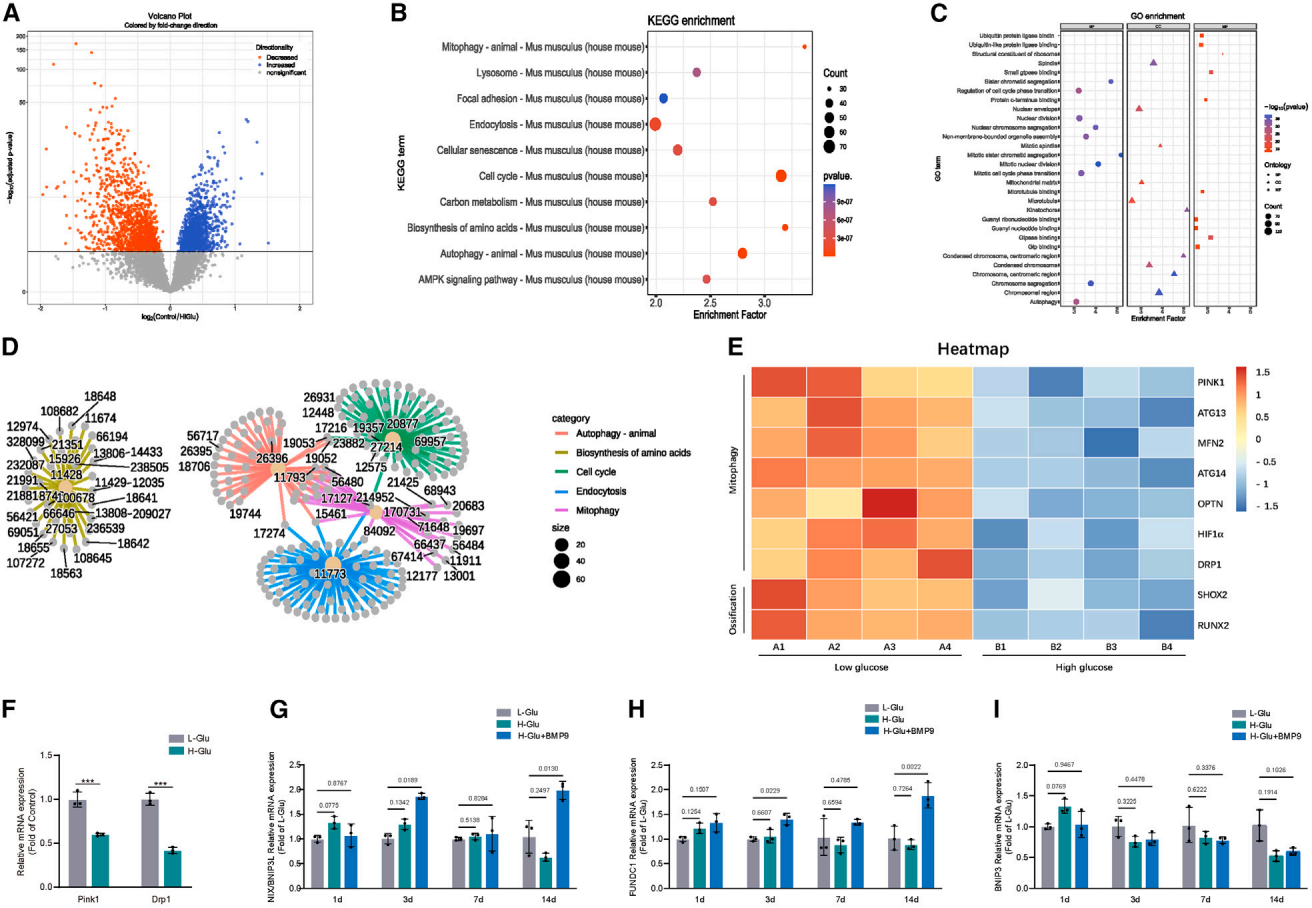
RNA-seq data analysis of differentially expressed genes in MC3T3-E1 cells cultured in L-Glu and H-Glu MC3T3-E1 cells were cultured in osteogenic medium for 14 days, then eukaryotic transcriptome sequencing was performed. (A) The volcano map showed significant differences in gene expression between the two groups. (B) The enrichment analysis of KEGG pathway based on the significantly different genes in the volcano map showed that the main pathways involved in the damage of osteoblasts by high sugar were mitochondrial autophagy, cell autophagy and cell cycle. (C) GO ontology functional enrichment showed that the main pathways involved in the damage of osteoblasts by high glucose were ubiquitin-proteasome system, ubiquitin-proteasome system, and mitochondrial autophagy. (D) The KEGG pathway loop map showed the main mechanisms of hyperglycemic damage: mitochondrial autophagy, autophagy, and cell cycle. (E) Genes of interest with significant differences. *n* = 4 biological replicates. (F) MC3T3-E1 cells were cultured in osteogenic medium for 14 days. Expressions of PINK1 and Drp1 were determined by RT-qPCR. (G–I) MC3T3-E1 cells were cultured in osteogenic medium for 1, 3, 7, and 14 days. Expressions of marker genes involved in ubiquitin-dependent mitophagy pathway at different time points were determined by RT-qPCR. Data presented as mean ± SD. *n* = 3 biological replicates. One-way ANOVA was used for comparison among multiple groups. A t test was used for comparison between two groups. ∗*p* < 0.05; ∗∗*p* < 0.01; ∗∗∗*p* < 0.001. Ns, no significance.

The aforementioned results demonstrated that high glucose level inhibited the late stage of osteogenic differentiation and impaired mitochondrial function of these cells. Then, what is its molecular mechanism? To address this question, MC3T3-E1 cells cultured in low glucose or high glucose osteoblastogenic induction media for 14 days were examined by RNA sequencing (RNA-seq) analysis (Figure 3A). Kyoto Encyclopedia of Genes and Genomes (KEGG) pathway enrichment analysis showed that the major pathways involved in the differential genes between the two groups were mitophagy, autophagy, and cell cycle (Figures 3B and 3D). Gene ontology (GO) functional enrichment showed that the major pathways involved in the differentially expressed genes were ubiquitin proteasome system and mitophagy (Figure 3C). As shown in Figure 3E, further analysis of mitophagy genes revealed that transcription levels of PINK1 and dynamic-associated protein 1 (Drp1) were significantly reduced in H-Glu group compared to L-Glu group. We further verified the expression of Drp1 and PINK1 by qPCR (Figure 3F). It is worth mentioning that we also detected mitophagy marker genes of non-ubiquitination-dependent pathways by qPCR and there were no significantly difference between the two groups (Figures 3G–3I). These findings suggest that marker gene PINK1 of mitophagy signaling pathway and Drp1 may be important factors involved in the mechanism by which high glucose inhibits osteogenic differentiation.

### High glucose inhibited PINK1/Drp1 signaling, leading to impaired osteogenic differentiation

**Figure 4:**
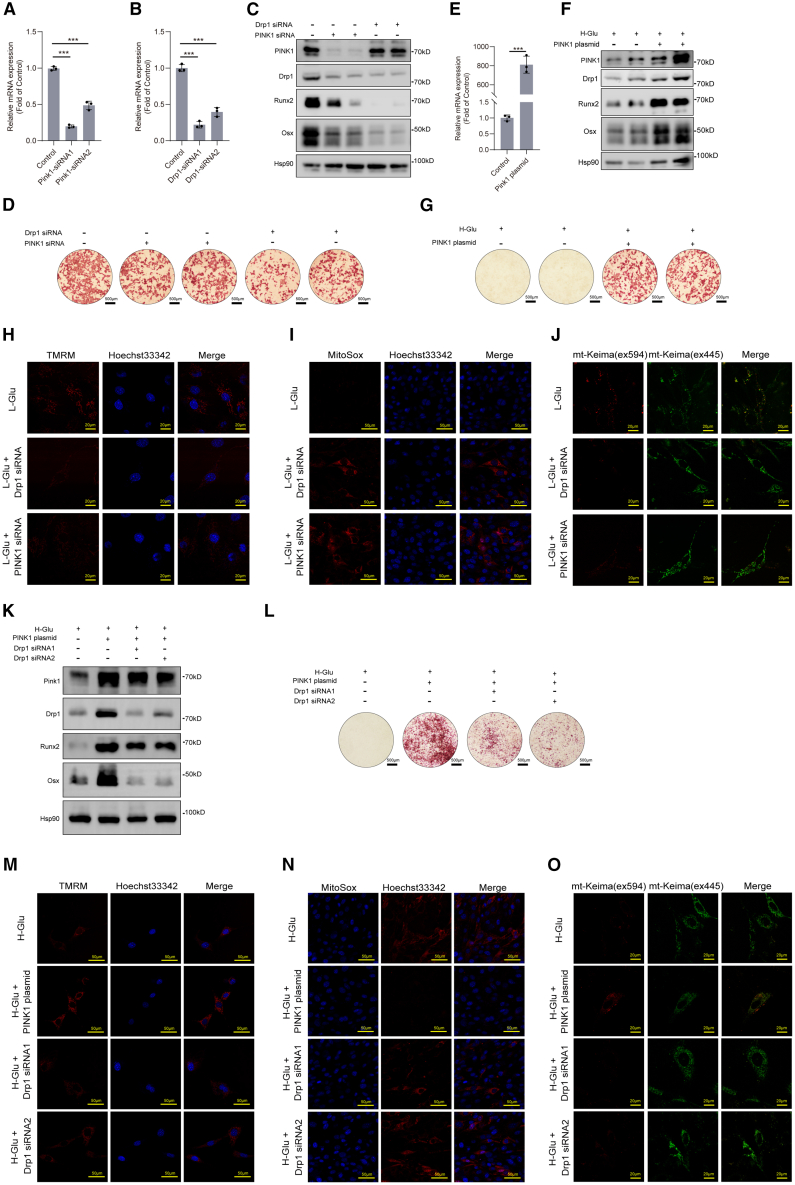
High glucose inhibited PINK1/Drp1 signaling, leading to impaired osteoblastogenic differentiation (A and B) Expressions of PINK1 and Drp1 genes were determined by RT-qPCR after siRNA transfection. (C) MC3T3-E1 cells were cultured in osteogenic medium (L-Glu) for 14 days after siRNA transfection. Expressions of PINK1, Drp1, Runx2, and Osx were determined by WB. (D) MC3T3-E1 cells were cultured in osteogenic medium (L-Glu) for 21 days after siRNA transfection. Alizarin red S staining was performed. Scale bars, 500 μm. (E) Expression of PINK1 gene was determined by RT-qPCR after PINK1 plasmid transfection. (F) MC3T3-E1 cells were cultured in osteogenic medium (H-Glu) for 14 days after plasmid transfection. Expressions of PINK1, Drp1, Runx2, and Osx were determined by WB. (G) MC3T3-E1 cells were cultured in osteogenic medium (H-Glu) for 21 days after plasmid transfection. Alizarin red S staining was performed. Scale bars, 500 μm. (H) MC3T3-E1 cells were cultured in osteogenic medium (L-Glu) for 14 days after siRNA transfection. MMP of living cells was assessed by the Image-iT TMRM Reagent. Scale bars, 20 μm. (I) MC3T3-E1 cells were cultured in osteogenic medium (L-Glu) for 14 days after siRNA transfection. Mitochondrial reactive oxygen species of living cells were assessed by the MitoSox Reagent. Scale bars, 50 μm. (J) MC3T3E1 cells were transfected with the mito-Keima plasmid and cultured in osteogenic medium (L-Glu) for 14 days. The fluorescent dots of mito-Keima were observed by confocal microscopy. Scale bars, 20 μm. (K) MC3T3-E1 cells were cultured in osteogenic medium (H-Glu) for 14 days after plasmid transfection and siRNA transfection. Expression of PINK1, Drp1, Runx2, and Osx were determined by WB. (L) MC3T3-E1 cells were cultured in osteogenic medium (H-Glu) for 14 days after plasmid transfection and siRNA transfection. Alizarin red S staining was performed. Scale bars, 500 μm. (M) MC3T3-E1 cells were cultured in osteogenic medium (H-Glu) for 14 days after plasmid transfection and siRNA transfection. MMP of living cells was assessed by the Image-iT TMRM Reagent. Scale bars, 50 μm. (N) MC3T3-E1 cells were cultured in osteogenic medium (H-Glu) for 14 days after plasmid transfection and siRNA transfection. Mitochondrial reactive oxygen species of living cells were assessed by the MitoSox Reagent. Scale bars, 50 μm. (O) MC3T3-E1 cells were cultured in osteogenic medium (H-Glu) for 14 days after plasmid transfection and siRNA transfection. The fluorescent dots of mito-Keima were observed by confocal microscopy. Scale bars, 50 μm. Data presented as mean ± SD. A t test was used for comparison between two groups. One-way ANOVA was used for comparison among multiple groups. ∗*p* < 0.05; ∗∗*p* < 0.01; ∗∗∗*p* < 0.001. Ns, no significance.

To further validate the functionality of PINK1 and Drp1 in osteogenic differentiation, we inhibited the expression of PINK1 and Drp1 in MC3T3-E1 cells by siRNA. Figures 4A and 4B demonstrated that the expression of PINK1 and Drp1 genes was significantly knocked down. We substantiated the involvement of PINK1/Drp1 signaling in the process of osteogenic differentiation through gene knockdown under normal glucose culture. Evidently, the targeted suppression of PINK1 or Drp1 significantly inhibited the expression levels of Runx2 and Osx (Figure 4C). We also observed that inhibiting the expression of PINK1 resulted in the downregulation of Drp1 protein. Conversely, inhibiting Drp1 did not affect the level of PINK1 protein, implying that PINK1 acts as the upstream signaling molecule of Drp1 (Figure 4C). Consistently, the quantification of mineralized nodules within osteoblasts, following the downregulation of PINK1 and Drp1, exhibited a substantial reduction compared to the control group (Figure 4D).

We further constructed an overexpressed PINK1 plasmid and transfected osteoblasts. Then, we induced osteogenic differentiation of MC3T3-E1 cells in high glucose medium. Figures 4E and 4F showed that the expression of PINK1 gene and protein were significantly increased after transfection. Notably, this augmentation in PINK1 expression coincided with noteworthy enhancements in the expressions of Runx2 and Osx (Figure 4F). Alizarin red staining showed that the number of mineralized nodules of osteoblasts overexpressing PINK1 increased significantly under high glucose medium culture (Figure 4G).

We also examined the mitochondrial function of osteoblasts that had knocked down PINK1 and Drp1, respectively. In Figure 4H, TMRM staining was performed on living cells in each group to detect MMP. The results showed that inhibiting the expression of either Drp1 or PINK1 could decrease the MMP of cells. In Figure 4I, MitoSox was used to detect mitochondrial reactive oxygen species, and the results showed that knockdown of Drp1 or PINK1 could increase the superoxide level of cellular mitochondria. In Figure 4J, MCT3E1 cells were transfected with pMito-Keima to detect mitophagy, and the results showed that mitophagy was weakened in cells with the Drp1 or PINK1 knocked down.

To verify Drp1 is a downstream target of PINK1, we further knocked down Drp1 in the presence of overexpression of PINK1 in MCT3E1 cells and examined the osteogenic phenotype and mitochondrial function of osteoblasts in high glucose medium. Figures 4K and 4L showed that the protein levels of Runx2 and Osterix of cells in high glucose medium were significantly increased when PINK1 overexpressed, and the formation of mineralized nodules was increased; while Drp1 depletion significantly weakened this phenotype. Correspondingly, the MMP decreased and the level of mitochondrial superoxide of PINK1 overexpressed cells in high glucose medium increased significantly after Drp1 knockdown (Figures 4M and 4N). Furthermore, the level of mitophagy of PINK1-overexpressed cells in high glucose medium was also significantly decreased after Drp1 was knocked down (Figure 4O). Collectively, these findings indicate that the PINK1/Drp1 signaling exerts positive influence on mitochondrial function of osteoblasts and osteogenic differentiation. Overexpression of PINK1 can rescue the impaired osteogenic differentiation under high glucose medium.

### BMP9 reversed both osteogenic differentiation impairment and mitochondrial dysfunction induced by high glucose in MC3T3-E1 cells

**Figure 5:**
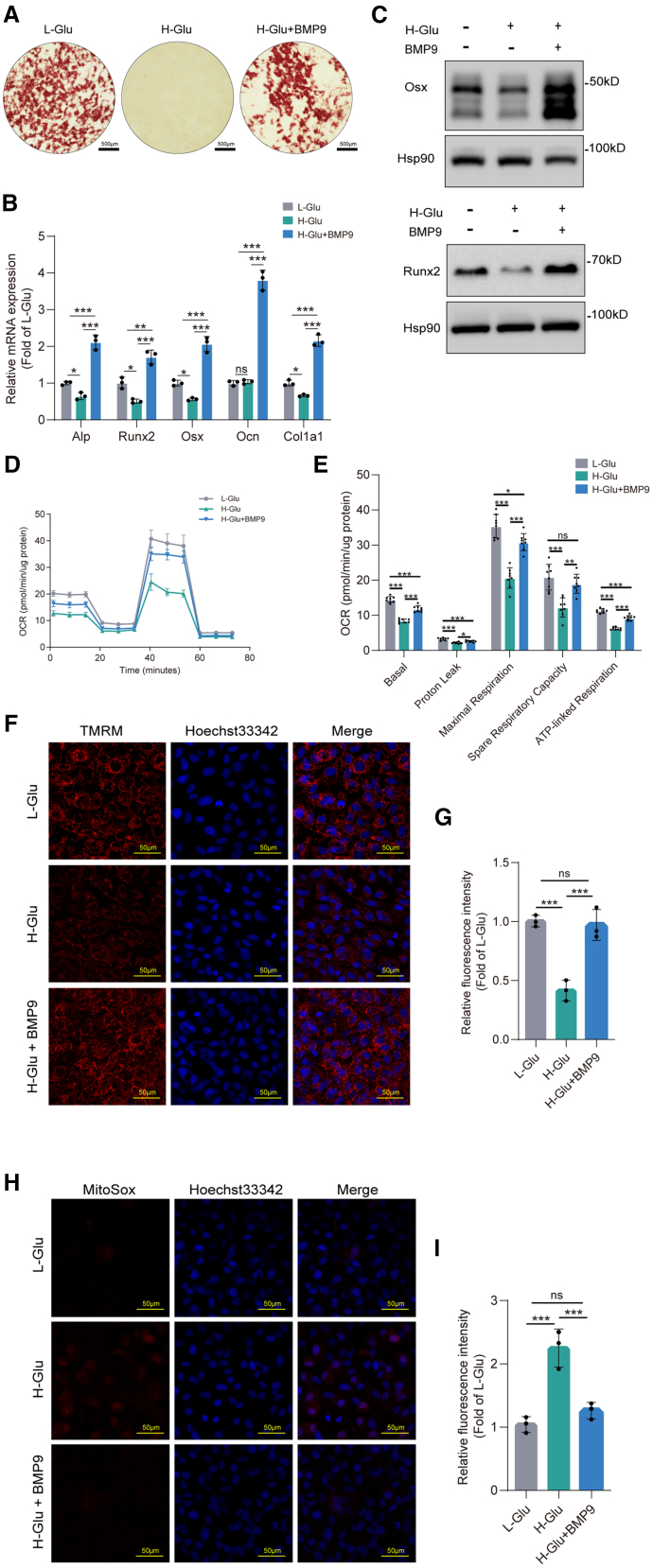
BMP9 reversed both the osteoblastogenic differentiation impairment and mitochondrial dysfunction induced by high glucose in MC3T3-E1 cells The concentration of glucose in the medium of the L-Glu group was 5.5 mmol/L, and that in the medium of the H-Glu and H-Glu+BMP9 group was 25 mmol/L. The final concentration of BMP9 was 100 ng/ml. (A) Alizarin red S staining was performed in MC3T3-E1 cells after 21 days of osteogenic induction. Scale bars, 500 μm. (B) MC3T3-E1 cells were cultured in osteogenic medium for 14 days. Expressions of osteogenic markers were determined by RT-qPCR. (C) MC3T3-E1 cells were cultured in osteogenic medium for 14 days. Expressions of Runx2 and Osx were determined by WB. (D and E) MC3T3-E1 cells were cultured in osteogenic medium for 14 days. Mitochondrial oxidative capacity was measured in real time. Basal respiration, ATP-linked respiration, maximal respiration, and spare respiratory capacity were calculated by WAVE software. (F and G) MC3T3-E1 cells were cultured in osteogenic medium for 14 days. MMP of living cells was assessed by the Image-iT TMRM Reagent. Scale bars, 50 μm. (H and I) MC3T3-E1 cells were cultured in osteogenic medium for 14 days. Mitochondrial reactive oxygen species of living cells were assessed by the MitoSox Reagent. Scale bars, 50 μm. Data presented as mean ± SD. *n* = 3 biological replicates. One-way ANOVA was used for comparison among multiple groups. ∗*p* < 0.05; ∗∗*p* < 0.01; ∗∗∗*p* < 0.001. Ns, no significance.

The aforementioned results show that high glucose level inhibits osteogenic differentiation and impairs mitochondrial function of MC3T3-E1 cells, so we are interested in whether there are molecules that can protect the mitochondrial function and promote osteogenic differentiation of MC3T3-E1 cells exposed to high glucose level. Our previous mice studies have demonstrated that bone morphogenetic protein 9 (BMP9) is effective in improving both ovariectomized (OVX) and senile osteoporosis.^31^^,^^32^ In this study, an obvious less mineralized nodule formation was observed in H-Glu group compared to L-Glu group. However, the impaired osteogenic differentiation induced by high glucose treatment was partially reversed by BMP9 treatment: mineralized nodule formation was increased after BMP9 treatment relative to L-Glu group (Figure 5A). Moreover, expression levels of ALP, Runx2, Osx, Ocn, and Col1a1 were significantly increased after BMP9 treatment (Figure 5B). Consistently, the WB results showed that the protein levels of Osx and Runx2 were increased after BMP9 treatment (Figure 5C).

We used extracellular flux analysis to real-time monitor mitochondrial respiratory capacity *in vitro*. BMP9 treatment dramatically increased basal, ATP production-coupled respiration, maximal respiration, and the spare respiratory capacity (Figures 5D and 5E). Furthermore, the result of TMRM signal showed that the MMP was significantly increased after BMP9 treatment (Figures 5F and 5G). We also examined superoxide in the mitochondria of live MC3T3-E1 cells using mitochondrial superoxide indicator MitoSOX Red. In the MC3T3-E1 cells cultured in high glucose, superoxide production was higher than that in control cells, and BMP9 significantly reduced superoxide production (Figures 5H and 5I). Collectively, these data indicate that BMP9 could improve mitochondrial function of MC3T3-E1 cells under high glucose medium culture.

### BMP9 activated PINK1/Drp1-mediated mitophagy, leading to the reversal of high glucose-induced osteogenic differentiation impairment in MC3T3-E1 cells

**Figure 6:**
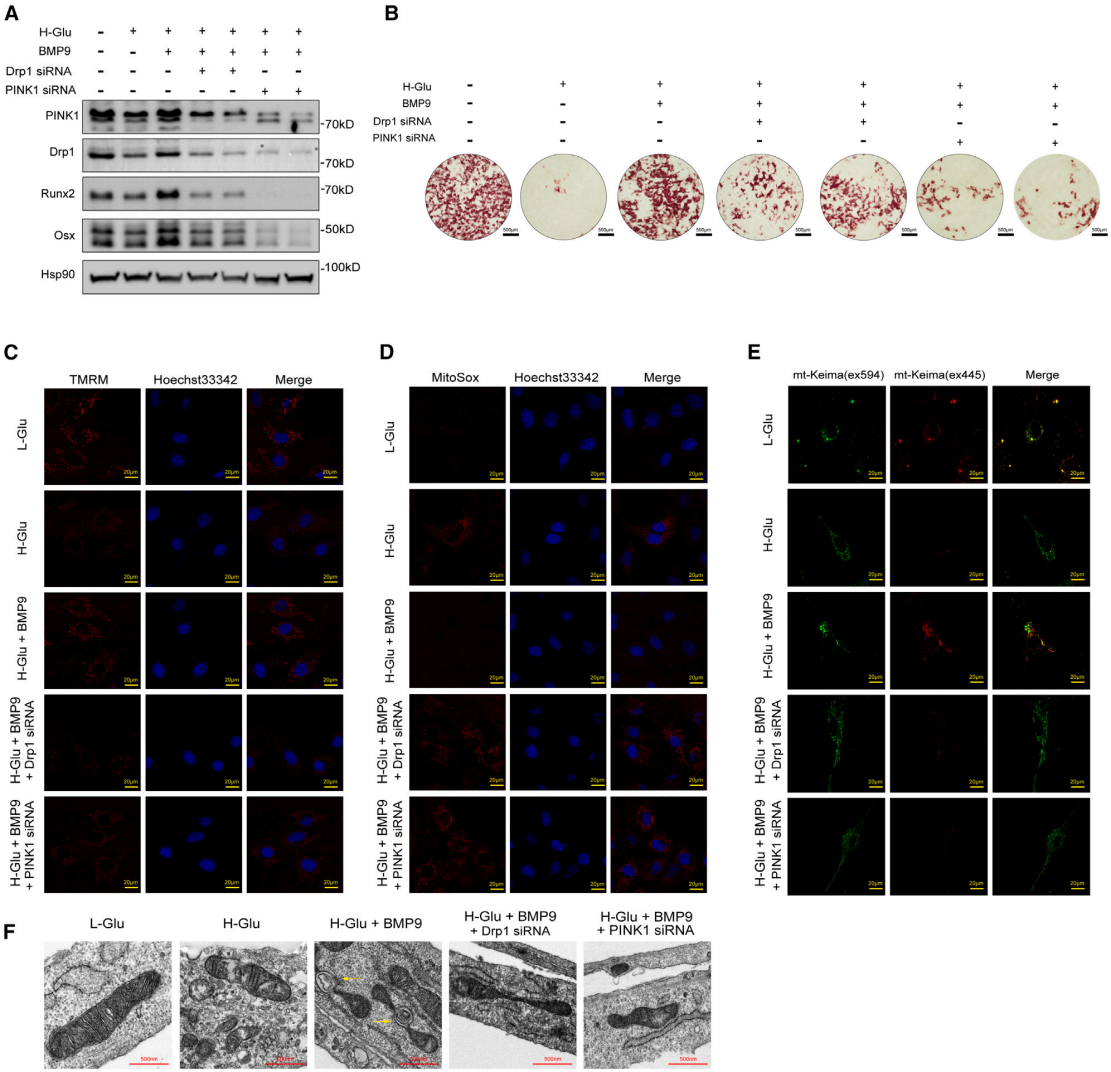
BMP9 activated PINK1/Drp1-mediated mitophagy, leading to the reversal of high glucose-induced osteoblast differentiation impairment in MC3T3-E1 cells The concentration of glucose in the medium of the L-Glu group was 5.5 mmol/L, and that in the medium of the H-Glu, H-Glu+BMP9, Drp1 siRNA and PINK1 siRNA groups was 25 mmol/L. The final concentration of BMP9 in H-Glu+BMP9, Drp1 siRNA and PINK1 siRNA groups was 100 ng/ml. (A) MC3T3-E1 cells were cultured in osteogenic medium for 14 days after siRNA transfection. Expressions of PINK1, Drp1, Runx2, and Osx were determined by WB. (B) MC3T3-E1 cells were cultured in osteogenic medium for 21 days after siRNA transfection. Alizarin red S staining was performed. Scale bars, 500 μm. (C) MC3T3-E1 cells were cultured in osteogenic medium for 14 days after siRNA transfection. MMP of living cells was assessed by the Image-iT TMRM Reagent. Scale bars, 20 μm. (D) MC3T3-E1 cells were cultured in osteogenic medium for 14 days after siRNA transfection. Mitochondrial reactive oxygen species of living cells were assessed by the MitoSox Reagent. Scale bars, 20 μm. (E) MC3T3E1 cells were transfected with mito-Keima plasmid and siRNA and cultured in osteogenic medium for 14 days. The fluorescent dots of mito-Keima were observed by confocal microscopy. Scale bars, 20 μm. (F) Representative electron micrographs show mitochondria and mitophagosomes (arrowhead) in MC3T3-E1 cells of different groups. Scale bars, 500 nm. Data presented as mean ± SD. A t test was used for comparison between two groups. One-way ANOVA was used for comparison among multiple groups. ∗*p* < 0.05; ∗∗*p* < 0.01; ∗∗∗*p* < 0.001. Ns, no significance.

To explore the regulatory mechanism of BMP9 on mitophagy, we inhibited the expression of PINK1 and Drp1 in cells by siRNA. After Drp1 or PINK1 depletion, the protein levels of Runx2 and Osterix of cells in H-Glu+BMP9 group were significantly decreased (Figure 6A), and the formation of mineralized nodules was inhibited, as compared with only treated with BMP9 (Figure 6B). Correspondingly, after PINK1 or Drp1 knockdown, the MMP decreased, and the level of mitochondrial superoxide of cells in H-Glu+BMP9 group increased significantly (Figures 6C and 6D). Furthermore, the level of mitochondrial autophagy of cells in H-Glu+BMP9 group was also significantly decreased after Drp1 or PINK1 was knocked down (Figure 6E).

We further observe the ultrastructure of mitochondria of MC3T3-E1 cells by transmission electron microscope. Figure 6F showed mitochondria of L-Glu cells showed a bilayer-membrane rod-like structure with clear mitochondrial ridge. Mitochondria of cells in H-Glu group were swollen, and mitochondrial ridge structure was destroyed and disappeared. The ridge structure of some mitochondria in H-Glu+BMP9 group was blurred, and the damaged mitochondria could be seen to phagocytosed and degraded by double-layer membrane structure (Figure 6F). While mitochondrial membrane structure was damaged but mitochondrial autophagy was not observed in PINK1 or Drp1 knockdown groups.

Our results indicate that BMP9 effectively ameliorates osteogenic differentiation of cells under high glucose condition by activating PINK1/Drp1 signaling axis.

### BMP9 activated PINK1/Drp1-mediated mitophagy, leading to the reversal of high glucose-induced osteogenic differentiation impairment in BMSCs

**Figure 7:**
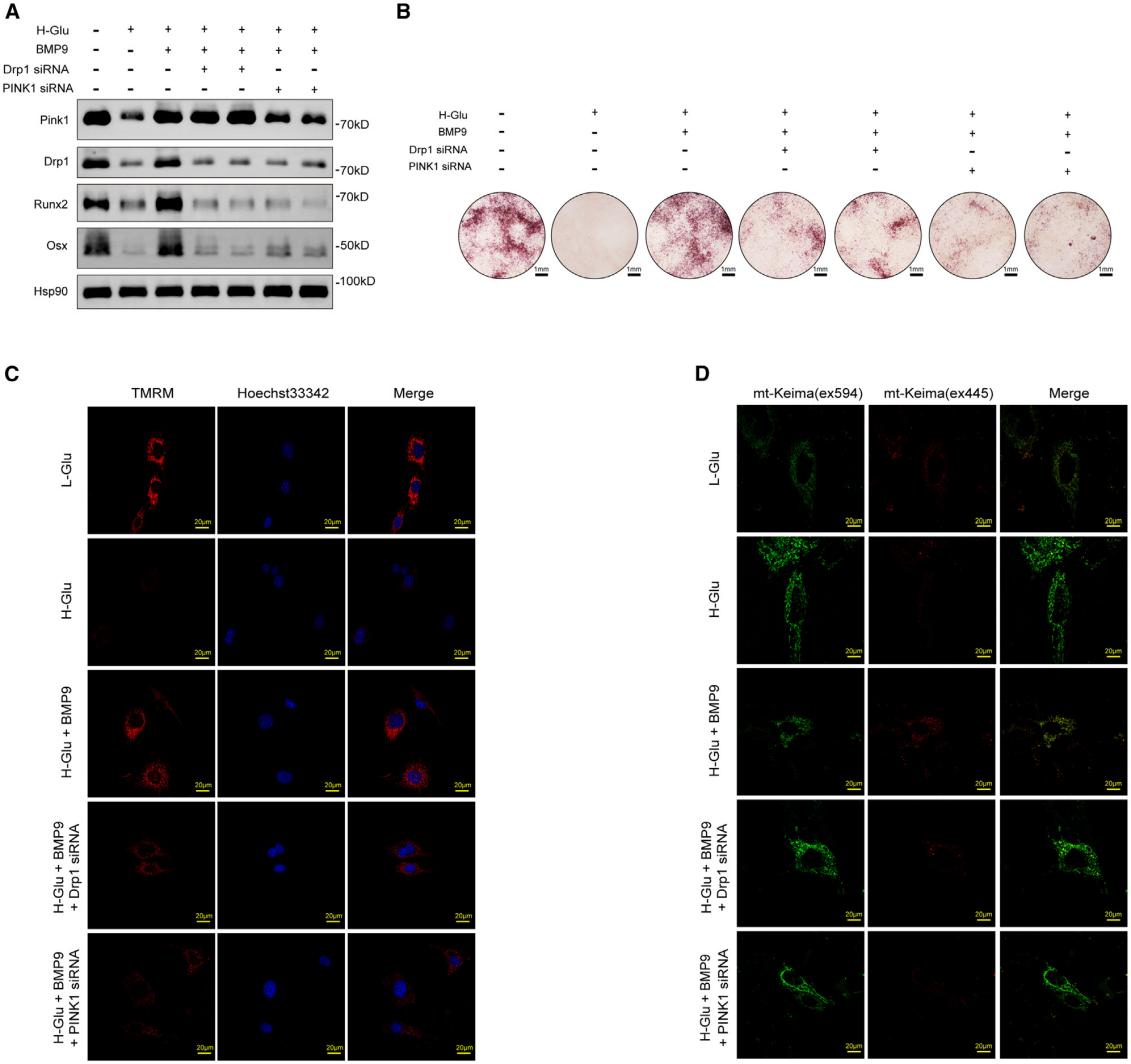
BMP9 activated PINK1/Drp1-mediated mitophagy, leading to the reversal of high glucose-induced osteoblast differentiation impairment in BMSCs The concentration of glucose in the medium of the L-Glu group was 5.5 mmol/L, and that in the medium of the H-Glu, H-Glu+BMP9, Drp1 siRNA and PINK1 siRNA groups was 25 mmol/L. The final concentration of BMP9 in H-Glu+BMP9, Drp1 siRNA and PINK1 siRNA groups was 100 ng/ml. (A) BMSCs cells were cultured in osteogenic medium for 14 days after siRNA transfection. Expressions of PINK1, Drp1, Runx2, and Osx were determined by WB. (B) BMSCs cells were cultured in osteogenic medium for 21 days after siRNA transfection. Alizarin red S staining was performed. Scale bars, 1 mm. (C) BMSCs cells were cultured in osteogenic medium for 14 days after siRNA transfection. MMP of living cells was assessed by the Image-iT TMRM Reagent. Scale bars, 20 μm. (D) BMSCs cells were transfected with mito-Keima plasmid and siRNA and cultured in osteogenic medium for 14 days. The fluorescent dots of mito-Keima were observed by confocal microscopy. Scale bars, 20 μm.

We conducted experiments to explore whether high glucose inhibits osteogenic differentiation of bone marrow mesenchymal stem cells (BMSCs) through the mitophagy-associated PINK1/Drp1 signaling pathway and whether BMP9 has a reversal effect. After Drp1 or PINK1 depletion, the protein levels of Runx2 and Osterix of cells in H-Glu+BMP9 group were significantly decreased (Figure 7A), and the formation of mineralized nodules was inhibited, as compared with only treated with BMP9 (Figure 7B). Correspondingly, the MMP of cells in H-Glu+BMP9 group significantly decreased after PINK1 or Drp1 knockdown (Figure 7C). Furthermore, the level of mitophagy of cells in H-Glu+BMP9 group was also significantly decreased after Drp1 or PINK1 was knocked down (Figure 7D). The results indicate that BMP9 can also effectively ameliorate osteogenic differentiation of BMSCs under high glucose by augmenting the activity of the PINK1/Drp1 signaling axis.

### BMP9 treatment in diabetic mice enhances bone mineral density and improves bone microarchitecture

**Figure 8:**
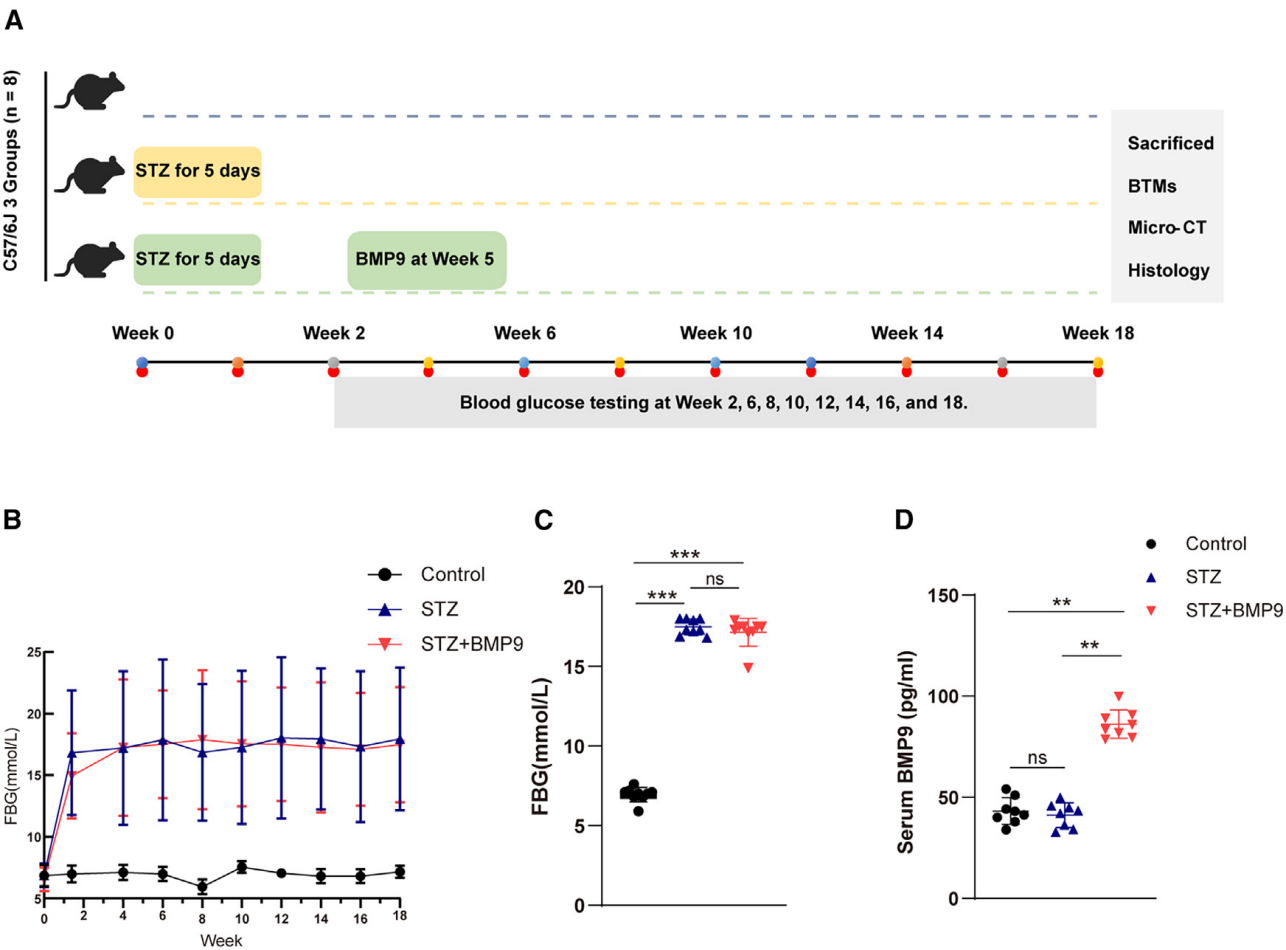
Diabetic mice models induced by STZ were treated with BMP9 (A) Flow charts for the establishment of STZ mice model. (B) Fasting blood glucose concentrations were measured and compared every two weeks from week 2 to week 18. (C) Fasting blood glucose concentrations at week 18 were compared between groups. (D) Serum BMP9 levels were detected at week 18. Data presented as mean ± SD. *n* = 8 biological replicates. One-way ANOVA was used for comparison among multiple groups. ∗*p* < 0.05; ∗∗*p* < 0.01; ∗∗∗*p* < 0.001. Ns, no significance.

To further verify the effects of BMP9 on bone mineral density (BMD) and bone quality *in vivo*, we constructed an streptozotocin (STZ) induced diabetes mice model. The design of the animal experiment is shown in Figure 8A. All diabetic mice remained hyperglycemic until the end of the study. Although serum BMP9 concentration was higher in STZ+BMP9 group, blood glucose was similar between STZ and STZ+BMP9 group. (Figures 8B–8D).

**Figure 9:**
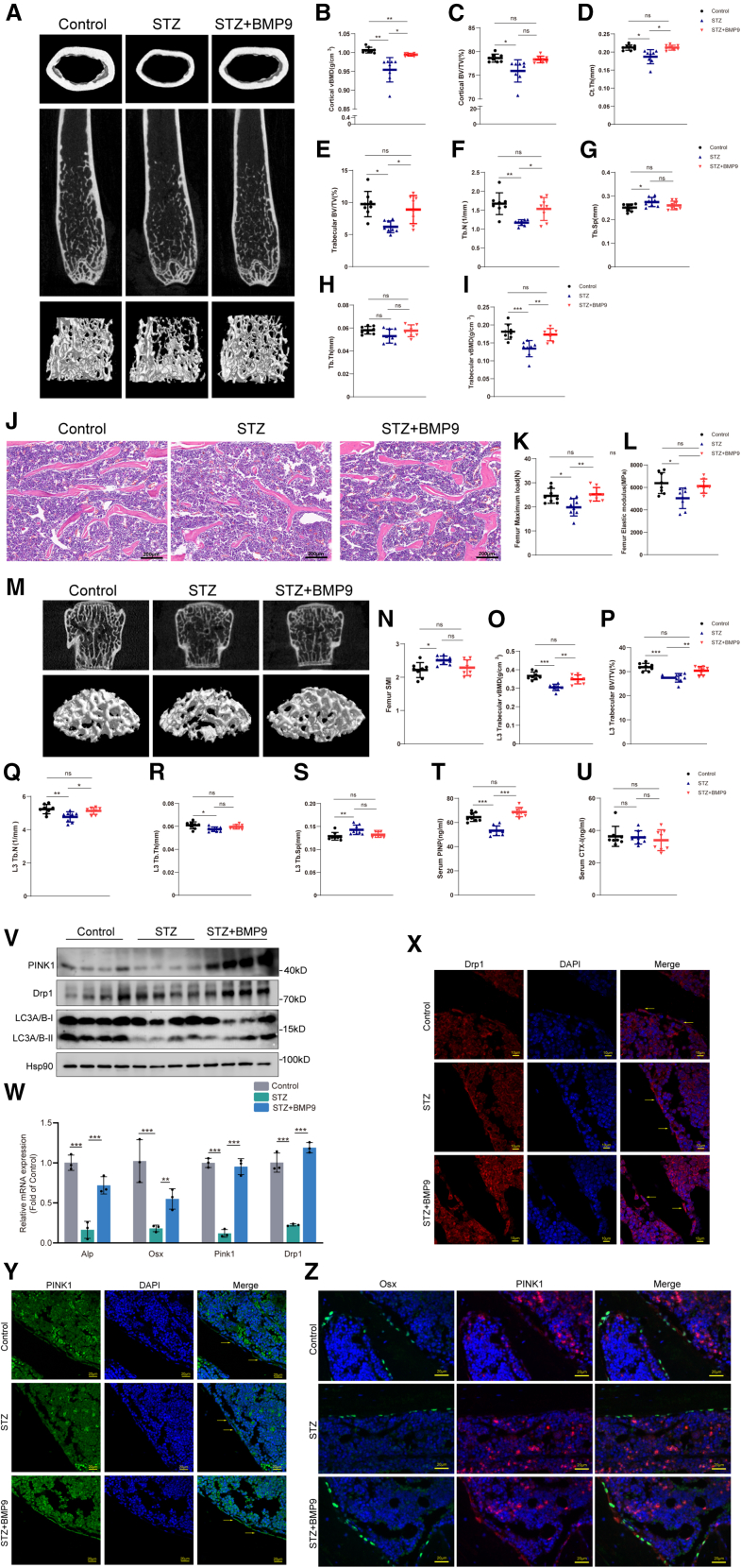
BMP9 promoted mitophagy markers to the control levels in diabetic mice in vivo (A) Representative images derived from micro-CT analysis, including 2D image construction of distal femur, 3D images reconstruction of trabecular bone of distal femur, and 3D image reconstruction of the femoral midshaft corticoid bone. (B–D) Quantitative analysis of the vBMD, BV/TV and Ct.Th of corticoid bone by micro-CT. (E–I) Quantitative analysis of the BV/TV, Tb.N, Tb.Sp, Tb.Th, and vBMD of trabecular by micro-CT. (J) Representative images of HE-stained decalcified femur sections. Scale bars, 200 μm. (K–M) The right femur was isolated and subjected to biomechanical properties analysis. The maximum load, elastic modulus and SMI were evaluated for each group. (N) Representative images derived from micro-CT analysis, including 2D image construction and 3D image reconstruction of L3 lumbar vertebra. (O–S) Quantitative analysis of the vBMD, BV/TV, Tb.N, Tb.Th, and Tb.Sp of L3 by micro-CT. (T and U) The levels of serum bone turnover parameters PINP and CTX-I were detected by ELISA. (V) WB analysis of protein levels of mitophagy marker in skull. (W) Expressions of ALP, Osx, Drp1, and PINK1 in skull were determined by RT-qPCR. (X) Immunofluorescence analysis of Drp1 and PINK1 expression in femur sections. Scale bars, 10 μm. (Y) Immunofluorescence analysis of PINK1 expression in femur sections. Scale bars, 20 μm. (Z) Immunofluorescence results of double labeling of osteoblasts with PINK1 and Drp1 in femur sections. Scale bars, 20 μm. Data presented as mean ± SD. *n* = 8 biological replicates. One-way ANOVA was used for comparison among multiple groups. ∗*p* < 0.05; ∗∗*p* < 0.01; ∗∗∗*p* < 0.001. Ns, no significance.

As shown in Figure 9A, a 3D reconstruction of the cortical bone and trabecular bone at the distal femurs clearly showed thinner cortical bone and the breakage of cancellous bone in STZ diabetic mice, and the administration of BMP9 improved the cortical bone thickness and microarchitecture of the distal femur trabecular bone. Moreover, micro-CT analysis of the distal femur showed decreased cortical BMD in the STZ mice and improved BMD in the STZ+BMP9 group (*p* < 0.05) (Figure 9B). Other cortical bone parameters, including BV/TV and Ct.Th, were significantly decreased in STZ mice as compared with controls (*p* < 0.05). The Ct.Th value of STZ mice treated with BMP9 increased compared to that in the STZ group (*p* < 0.05) (Figure 9D), while the difference in BV/TV value between the two groups was not statistically significant (Figure 9C). Micro-CT analysis also showed that compared with mice in the control group, STZ mice exhibited obvious decrease in BV/TV, Tb.N, and BMD and increased Tb.Sp of the distal femur trabecular bone, while the BV/TV, Tb.N, and BMD of mice treated with BMP9 increased by 43% (*p* < 0.05), 30% (*p* < 0.05), and 29% (*p* < 0.01), respectively (Figures 9E–9I). Consistent with the micro-CT analysis results, H&E staining of the distal femur sections confirmed a reduction in trabecular number and trabecular thickness in STZ mice compared with that of the control, and these reductions were attenuated by BMP9 treatment (Figure 9J).

Furthermore, compared with the control group, the maximum load and Young’s modulus of femur in STZ group were significantly decreased (*p* < 0.05), the maximum load and elastic modulus of femur in STZ+BMP9 group were increased by 27.1% (*p* < 0.01), and the value of elastic modulus was increased by 21.6% compared with STZ mice, but there was no statistical difference. The structural simulation index was not significantly different from that of STZ group (Figures 9K and 9L).

Additionally, we examined the parameters of lumbar vertebrae in mice. The bone density and 3D reconstruction images of the third lumbar vertebra in mice were shown in Figure 9M. Compared with the normal control group, the lumbar-spine BMD in the STZ group decreased by 16.9% (*p* < 0.01) and increased by 14.1% (*p* < 0.01) in STZ+BMP9 group. The BV/TV, Tb.N, and Tb.Th values of lumbar cancellous bone in the STZ group were significantly decreased compared with the control group, and the Tb.Sp value was significantly increased in the STZ mice. Compared with the STZ group, the BV/TV and Tb.N values of lumbar cancellous bone in the STZ+BMP9 group were increased by 10.7% and 7.5%, respectively, and there was no significant difference compared with the control group. The Tb.Sp and Tb.Th values of lumbar vertebrae in STZ+BMP9 group were not significantly different from those in STZ group (Figures 9N–9S).

We then examined bone turnover markers in mice. Compared with the normal control group, the serum procollagen I N-terminal propeptide (PINP) level of mice in STZ group was significantly decreased (*p* < 0.01), but was significantly increased in STZ+BMP9 group compared with the STZ mice group (*p* < 0.01) (Figure 9T). There was no significant difference in serum C-terminal telopeptide of type 1 collagen (CTX-I) concentration between three groups (Figure 9U). These results suggest that BMP9 has the ability to ameliorate diabetic osteoporosis.

### BMP9 promoted mitophagy markers to the control levels in diabetic mice *in vivo*

The favorable bone effect of BMP9 in diabetic STZ mice was investigated mechanistically. The protein from parietal bone in mice was quantified by WB. The results demonstrated that the expressions of mitophagy related proteins PINK1 and Drp1 were significantly increased in mice treated with BMP9, and the ratio of autophagy marker proteins LC3A/BⅡ and LC3A/BⅠ were significantly increased in STZ+BMP9 group (Figure 9V). Moreover, qPCR results showed expression levels of ALP, Osx, Drp1, and PINK1 were significantly increased in BMP9 treatment group (Figure 9W). We further stained the bone sections of the femur in mice by immunofluorescence. As shown in Figure 9X, the expression of Drp1 protein in osteoblasts located on the bone trabecular surface of mice in STZ group was significantly decreased compared with that of control mice, and was significantly increased after BMP9 administration, which was consistent with the variation trend of PINK1 in Figure 9Y. In order to further confirm the aforementioned results, double-label immunofluorescence staining was performed on the femur section. Osx was used to label osteoblasts and the expression of PINK1 in osteoblasts was detected. Compared with the control group, the expression of PINK1 in osteoblasts in the STZ group was significantly decreased. The expression of PINK1 in osteoblasts in STZ+BMP9 group was significantly higher than that in STZ group (Figure 9Z). These results suggested that BMP9 can promote the expression of mitophagy protein in bone tissue of diabetic mice, promoting mitophagy may be an effective means to improve the bone mass and quality in diabetic mice.

## Discussion

In this study, we revealed that the suppressed PINK1/Drp1-mediated mitophagy is a molecular pathway responsible for impaired osteogenic differentiation in diabetes. Activation of mitophagy by BMP9 can mitigate osteoporosis induced by hyperglycemia.

Osteoblast-mediated bone formation requires enough ATP, which is generated through OXPHOS and glycolysis.^33^ Our research found that high glucose condition lasting to the late stage of differentiation could induce the decrease in basal and maximal respiration, ATP synthesis, and glycolytic rate of osteoblast. We also found mitochondrial dysfunctions, including the increased ROS, and decreased MMP in osteoblast cultured under high glucose level. Our RNA sequencing analysis suggested that impaired mitophagy might be the molecular mechanism for high glucose-induced inhibition of osteogenic differentiation.

Upon mitochondrial stress, mitophagy is activated and can selectively eliminate the damaged mitochondria, and thus stabilizing cellular homeostasis.^34^ Autophagy and mitophagy play a vital role in regulating osteoblasts’ proliferation and differentiation. Thus, we further explored the role of mitophagy in high glucose-induced inhibition of osteogenic differentiation and its related signaling pathways. PINK1 is an important promotor of mitochondrial autophagy. When MMP declines, the Ser228 and Ser402 groups of PINK1 will be phosphorylated,^35^ upon activation of PINK1 phosphorylation, ubiquitin on the mitochondrial outer membrane can be phosphorylated, then initialing mitophagy. We found that siRNA knockdown of PINK1 inhibited the ameliorative effect of BMP9 to impaired mitophagy and osteogenic differentiation in high glucose conditions, while overexpression of PINK1 can reverse the inhibitory effect of high glucose level on osteogenic differentiation.

Mitophagy and mitochondrial dynamics are close related. Drp1, a GTPase of the dynein superfamily, is the most important molecule in promoting mitochondrial fission. After Drp1 is recruited into the outer membrane of mitochondria, it will form helical oligomers, thereby inducing contraction and severing of the mitochondrial membrane.^36^ Our RNA sequencing analysis showed the decreased expression level of Drp1 in osteoblasts cultured in high glucose medium, suggesting the potential interplay between Drp1 and PINK1-induced mitophagy in impaired osteogenic differentiation caused by hyperglycemia. Then, we conducted a series of experiments to address the regulatory effect between Drp1 and PINK1. We found that the expression of Drp1 was downregulated after the knockdown of PINK1. In contrast, the expression of PINK1 did not change following the inhibition of Drp1, suggesting Drp1 as a downstream signaling molecule of PINK1. Our results also demonstrated that Drp1 knockdown can inhibit osteogenic differentiation and mitophagy and impair mitochondrial function. PINK1 was shown to directly phosphorylate S616 of Drp1 to regulate mitochondrial fission,^37^ which was consistent with our results. Taken together, we hypothesized that PINK1/Drp1-mediated mitophagy plays a critical role in osteogenic differentiation, and its suppression contributes to hyperglycemia-induced impaired osteoblastogenesis. To further verify this hypothesis, we then tried to find a molecule to activate this signaling pathway to maintain mitophagy of osteoblast in hyperglycemia and treat diabetic osteoporosis *in vivo*.

BMP9, a member of the transforming growth factor beta (TGF-β) superfamily, regulates various physiological processes such as angiogenesis, glucose metabolism, neurogenesis, and tumorigenesis. Our previous research found that BMP9 promotes osteoblastogenesis by upregulating LGR6 and activating the Wnt/β-catenin pathway and suppresses osteoclast differentiation by inhibiting the nuclear factor-κB (NF-κB) pathway.^31^ Our team’s other research also found that BMP9 reduces expression of senescent genes and alleviates senescence-associated secretory phenotype in bone microenvironment, simultaneously increasing bone mass and bone biomechanical properties in aged mice.^32^ As a continuation of work, we investigated the regulatory role of BMP9 on mitophagy in osteoblast and its protective effect on diabetic osteoporosis in this study. *In vitro*, we observed that BMP9 improved the osteogenic differentiation of MC3T3-E1 cells under high glucose conditions. This protective effect of BMP9 on osteoblasts could be mediated by the regulation of mitophagy through its capacity to upregulate PINK1/Drp1 expression. Whereas knockdowns of PINK1 and Drp1 can abolish the bone protective function of BMP9 under high glucose conditions.

Mitophagy dysfunction participates in pathogenesis diabetes and diabetes-related complications that include β-cell damage,^38^ glucose intolerance,^39^ cardiomyopathy,^40^ insulin resistance,^41^ and diabetes-associated cognitive dysfunction.^42^ Previous study has found that non-imprinted protein 2 in the Prader-Willi/Angelman syndrome region (NIPA2), a mitophagy-related molecule, was decreased in the bone tissue of diabetic mice.^43^ Our research found that PINK1/Drp1-mediated mitophagy was inhibited in the bone of diabetic mice and its activation by BMP9 ameliorated diabetic osteoporosis, indicating that PINK1/Drp1-mediated mitophagy may be a therapeutic target for diabetic osteoporosis. BMP9 is known to be a powerful promoter of osteogenic differentiation. Our research found that BMP9 rescued diabetic osteoporosis by addressing various suboptimal bone phenotypes, including promoting bone formation, improving bone density, enhancing the microstructure of cancellous and cortical bones, and ameliorating femur biomechanics. Notably, all these favorable bone effects of BMP9 in diabetes are not related to changes in blood glucose. These results suggest that BMP9 may act directly and specifically on bone tissue in diabetic mice, and this advantage makes BMP9 a potential therapeutic drug for diabetic osteoporosis.

In conclusion, our findings suggest the suppressed PINK1/Drp1-mediated mitophagy is responsible for the impairment of osteogenic differentiation in diabetes. BMP9 facilitates osteogenic differentiation and maturation under diabetic conditions through activating PINK1/Drp1-mediated mitophagy, ultimately exerting a bone-protective effect in diabetic mice. Further research on PINK1/Drp1-mitophagy pathway and its agonists, such as BMP9, is a promising way to explore the potential pathogenesis and therapeutic strategy for osteoporosis in diabetes.

### Limitations of the study

This study had several limitations. Firstly, the precise mechanism through which BMP9 activates PINK1/Drp1 signaling pathway requires further elucidation. Secondly, the late onset of skeletal phenotype in diabetic mice led to a limited number of extracted skull primary cells or mesenchymal stem cells with diminished activity. Thirdly, we did not use osteoblast conditional gene knockout mouse models to verify the impaired PINK1/Drp1-mediated mitophagy as a pathogenesis of diabetic osteoporosis.

## Resource availability

### Lead contact

Further information and requests for the resources and reagents should be directed to and will be fulfilled by the lead contact, Hong-yan Zhao (hyanzhao@163.com).

### Materials availability

This study did not generate any new unique reagents and components. The siRNA oligonucleotide sequences were provided in Table S1, and primer sequences of target genes were provided in Table S2.

### Data and code availability

• RNA sequencing data have been deposited at Zenodo and are publicly available as of the date of publication. Accession numbers are listed in the key resources table.
• This paper does not report the original code.
• Any additional information required to reanalyze the data reported in this work paper is available from the lead contact upon request.

## Acknowledgments

This research was supported by the 10.13039/501100001809National Natural Science Foundation of China (no. 81970758, 8230098, and 282070865) and Shanghai Sailing Program (no. 22YF1440100). We sincerely appreciate their generous funding, which made it possible for us to conduct this research project. We would also like to express our gratitude for the technical support provided by the laboratory staff at Shanghai National Clinical Research Center for Endocrine and Metabolic Diseases. Finally, we would like to thank the anonymous reviewers for their insightful and constructive feedback on our manuscript, which has significantly improved its overall quality.

## Author contributions

X.-j.C.: conceptualization, investigation, formal analysis, validation, writing – original draft, writing – review and editing. Y.-y.Y.: investigation, formal analysis, validation. Z.-c.P.: investigation, formal analysis, validation. J.-z.X.: investigation, validation. T.J.: investigation, validation. L.-l.Z.: investigation, validation. K.-c.Z.: methodology, writing – review and editing. D.Z.: methodology, writing – review and editing. J.-x.S.: methodology, writing – review and editing. C.-x.S.: methodology, writing – review and editing. L.-h.S.: methodology, writing – review and editing. B.T.: conceptualization, supervision, writing – review and editing, formal analysis. J.-m.L.: conceptualization, supervision, formal analysis, writing – review and editing. H.-y.Z.: conceptualization, supervision, formal analysis, funding acquisition, project administration, writing – review and editing.

## Declaration of interests

The authors declare no competing interests.

## STAR★Methods

### Key resources table

**Table:** 

REAGENT or RESOURCE	SOURCE	IDENTIFIER
**Antibodies**		

Mouse Anti-Hsp90 antibody	Santa Cruz	Cat# sc-13119
Rabbit Anti-PINK1 antibody	Novus	Cat# BC100-494S
Rabbit Anti-Drp1 antibody	Abcam	Cat# ab184247
Rabbit Anti-Runx2 antibody	CST	Cat# 8486
Rabbit Anti-Osx antibody	Abcam	Cat# ab209484
Anti-VDAC1/Porin antibody	Abcam	Cat# ab306581
LC3A/B antibody	CST	Cat# 4108
HRP-conjugated Alpha Tubulin antibody	Proteintech	Cat# HRP-66031
HRP-linked Anti-Mouse antibody	CST	Cat# 7076
HRP-linked Anti-Rabbit antibody	CST	Cat# 7074
CF 488 tyramide	Runnerbio China	Cat# Bry-880488
AF 647 tyramide	Runnerbio China	Cat# Bry-880647

**Chemicals, peptides, and recombinant proteins**		

α-modified minimal essential medium	Gibco	Cat# 32571101
Fetal bovine serum	Gibco	Cat# 10091148
Penicillin/streptomycin	Beyotime	Cat# C0222
β-glycerophosphate disodium salt hydrate	Sigma-Aldrich	Cat# 50020
L-ascorbic acid	Sigma-Aldrich	Cat# A4544
Lipofectamine 2000	Invitrogen	Cat# 11668027
Streptozotocin	Sigma-Aldrich	Cat# S0130
MitoSOX™ Red mitochondrial superoxide indicator	Invitrogen	Cat# M36008
Image-iT tetramethylrhodamine (TMRM) reagent	Invitrogen	Cat# I34361
Hoechst 33342	Invitrogen	Cat# H3570
Seahorse XF DMEM medium	Agilent	Cat# 103575–100
Hematoxylin Staining Solution	Beyotime	Cat# C0107
Super sensitive ECL luminescence reagent	Meilunbio	Cat# MA0186
Tri reagent	Sigma-Aldrich	Cat# T9424
Recombinant Mouse BMP-9	Biolegend	Cat# 553204

**Critical commercial assays**		

BCIP/NBT Alkaline Phosphatase Color Development Kit	Beyotime	Cat# C3206
Alizarin Red S Solution	Solarbio	Cat# G1450
Mitochondrial isolation kit	Thermo Scientific	Cat# 89874
Bicinchoninic acid (BCA) protein assay kit	Thermo Scientific	Cat# 23227
ROS assay kit	Beyotime	Cat# S0033S
ELISA Kit for Procollagen I N-Terminal Propeptide (PINP)	USCN Life Science	Cat# SEA957Hu
ELISA Kit for Cross Linked C-Telopeptide Of Type I Collagen (CTXI)	USCN Life Science	Cat# CEA665Hu
Mouse BMP-9 ELISA Kit	RayBiotech	Cat# Q9WV56
Seahorse XF Cell Mito Stress Test Kit	Agilent	Cat# 103015–100
PrimeScript reverse transcript master mix	TaKaRa	Cat# RR036Q
ChamQ Universal SYBR qPCR Master Mix	Vazyme	Cat# Q711-02
Total OXPHOS Rodent WB antibody cocktail	Abcam	Cat# ab110413
Bicinchoninic acid (BCA) protein assay kit	Thermo Scientific	Cat# 23227

**Deposited data**		

RNA sequencing data	This paper	Zenodo: https://doi.org/10.5281/zenodo.14174646

**Experimental models: Cell lines**		

MC3T3-E1 cells	ATCC	Cat# CRL-2593

**Experimental models: Organisms/strains**		

C57BL/6 mice	Charles River	Cat# 213

**Oligonucleotides**		

PINK1 siRNA	GenePharma	N/A
Drp1 siRNA	GenePharma	N/A
siRNA oligonucleotide sequences, see Table S1	This paper	N/A
Primer sequence of target genes in RT-qPCR, see Table S2	This paper	N/A

**Recombinant DNA**		

Mitochondria-targeted mKeima-Red expression plasmid	MBL	Cat# AM-V0251
PINK1 expression plasmid	GenePharma	N/A
Drp1 expression plasmid	Shanghai Xitubio Biotechnology	N/A
AAV-BMP9	Hanbio	N/A

**Software and algorithms**		

Zen software	Zeiss	N/A
R (version 4.3.2)	R Core Team	https://www.r-project.org
GraphPad Prism 9	GraphPad	https://www.graphpad.com/features

**Other**		

Instron 5569	Instron	https://www.instron.com/en/products/testing-systems/out-of-production-systems/electromechanical
SkyScan 1176	Bruker	https://www.accela.eu/bruker-biospin/skyscan-1176
Seahorse Extracellular Flux Analyzer XFe96	Agilent	https://www.agilent.com/cs/library/usermanuals/public/usermanual-xfe96-xf96-cell-characterization-cell-analysis-5994-0368en-agilent.pdf
QuantStudio Dx Real-Time PCR Instrument	Applied Biosystems	https://www.thermofisher.com/hk/en/home/clinical/diagnostic-testing/instruments-automation/genetic-analysis-instruments/quantstudio-dx-systems.html
NanoDrop ND-2000 spectrophotometer	Thermo Scientific	Cat# ND-2000C
eBlot Touch Imager	eBlot	https://www.e-blot.com/
JEM-1400Flash electron microscope	JEOL	https://www.jeol.com/products/scientific/tem/JEM-1400Flash.php
TissueFAXS system	TissueGnostics, Austria	https://tissuegnostics.com/products/scanning-and-viewing-software/tissuefaxs-imaging-software

### Experimental model and study participant details

#### Cell line

The preosteoblast line MC3T3-E1 cells (American Type Culture Collection) were cultured in α-modified minimal essential medium (α-MEM, Gibco) supplemented with 10% fetal bovine serum (FBS, Gibco) and 1% penicillin/streptomycin (Beyotime) at 37°C with 5% CO2. The culture medium was changed every two days.

BMSCs were isolated from the bone marrow of mice. First, mice were euthanized, followed by the dissection of its tibia, femur, and humerus. The ends of the bones were cut off with sharp scissors. The bone marrow was then flushed out from the bone cavities using α-MEM containing 10% FBS. The aspiration of bone marrow was filtered through a 70-micron mesh, then centrifuged, washed, and resuspended in α-MEM containing 10% FBS. The culture dishes were placed at 37°C in a 5% CO2 incubator. Non-adherent cells were removed after 24–72 h by changing the culture medium. Once the culture reached 70–90% confluence, the cells were subcultured at a 1:3 split ratio.

#### Mouse strain

Male C57BL/6 mice (Charles River), 9 weeks old, were maintained at the Experimental Animal Facility with temperatures of 22°C–24°C, humidity levels of 55%–60%, and a 12-h light/dark cycle. They were provided with water and a standard rodent chow diet. All animal experiments were conducted according to the guidelines for the humane use and care of laboratory animals and were approved by the Shanghai Jiao Tong University School of Medicine Animal Study Committee.

### Method details

#### Osteoblastogenic induction and bone morphogenetic protein 9 intervention

Osteoblastogenic induction medium consisted of α-MEM (Gibco) supplemented with 10% FBS (Gibco), 1% penicillin/streptomycin (Beyotime), 10mM β-glycerophosphate disodium salt hydrate (Sigma-Aldrich), and 50μM L-ascorbic acid (Sigma-Aldrich). The osteoblastogenic induction media were formulated with two concentrations of glucose: low glucose (5.5 mmol/L) and high glucose (25 mmol/L). BMP9 intervention was achieved through the supplementation of 100 ng/mL BMP9 (Biolegend) in the osteoblastogenic induction media. MC3T3-E1 cells or BMSCs were cultured in the osteoblastogenic induction media to induce osteogenic differentiation. Based on the glucose concentrations of the media and BMP9 intervention during osteogenic differentiation, the MC3T3-E1 cells or BMSCs were assigned to three groups: the low-glucose group (L-Glu), the high-glucose group (H-Glu), and the high-glucose group supplemented with BMP9 (H-Glu + BMP9).

#### Small interfering RNA (siRNA) and plasmid transfection

We purchased commercial and customized siRNAs and plasmids: a PINK1 siRNA (GenePharma), a Drp1 siRNA (GenePharma), a mitochondria-targeted mKeima-Red expression plasmid (pMT-mKeima-Red, MBL), and a PINK1 expression plasmid (GenePharma). MC3T3-E1 cells were transiently transfected with siRNA or plasmids using Lipofectamine 2000 (Invitrogen), following the transfection protocol provided by the Lipofectamine 2000 reagent. The siRNA oligonucleotide sequences were listed in Table S1.

#### Alp staining assay and Alizarin red staining assay

Alp staining of differentiated MC3T3-E1 cells was performed using a 5-bromo-4-chloro-3-indolyl phosphate/tetranitro blue tetrazolium chloride (BCIP/NBT) alkaline phosphatase color development kit (Beyotime) according to the manufacturer’s instructions. In brief, MC3T3-E1 cells were washed and fixed with 4% paraformaldehyde following the 7-day differentiation induction, and the staining solution was applied for 15 min at room temperature. Level of differentiation was quantified by visual examination of cells stained purple. Alizarin red S staining assay (Solarbio) was performed after 14 and 21 days of osteoblastic induction according to the manufacturer’s instructions. In brief, MC3T3-E1 cells were fixed with paraformaldehyde for 10 min. The mineralized nodules were stained with 0.2% Alizarin red and washed three times with PBS.

#### Western blot and qPCR analyses

Total protein was extracted by radioimmunoprecipitation (RIPA) lysis buffer (Biocolors, Shanghai, China) supplemented with Protease Inhibitor Cocktail (APExBIO, Houston, USA). Mitochondrial protein fraction was extracted using a mitochondrial isolation kit (Thermo Scientific) for cultured cells according to the manufacturer’s instruction. The concentration of protein was measured using a Bicinchoninic acid protein assay kit (Thermo Scientific), then the degenerated protein lysates were separated using sodium dodecyl sulfate–polyacrylamide gel electrophoresis (SDS-PAGE) for electrophoresis and then transferred to polyvinylidene difluoride (PVDF) membranes (Millipore). Membranes were blocked in 5% fat-free milk (Sangon Biotech) for 1.5 h at room temperature and then incubated overnight at 4°C with the primary antibodies: mouse anti-Hsp90, rabbit anti-PINK1, rabbit anti-Drp1, rabbit anti-Runx2, rabbit anti-Osx, anti-VDAC1/Porin antibody, LC3A/B antibody and antibodies of OXPHOS complexes I–V from total OXPHOS Rodent WB antibody cocktail (Abcam). HRP-conjugated secondary antibody incubation was performed at room temperature for 1 h. Immunoblotting signals were detected by super sensitive ECL luminescence reagent (Meilunbio, China) and visualized by the eBlot Touch Imager (eBlot, China).

For RNA extraction, total RNA was extracted from tissues or cultured cells using Tri reagent (Sigma-Aldrich). The absorbance ratio at 260/280 nm and the RNA concentration of all samples were detected using a NanoDrop ND-2000 spectrophotometer (Thermo Scientific). Reverse transcription was performed using the PrimeScript RT Master Mix (TaKaRa), and RT-qPCR was conducted using the ChamQ Universal SYBR qPCR Master Mix (Vazym). The measurements were taken using the QuantStudio Dx Real-Time PCR Instrument (Applied Biosystems, USA). The ΔΔCT method was used to evaluate the relative mRNA expression that was normalized to 36b4. Primer sequences were listed in Table S2.

#### Cell bioenergetic profiling

Mitochondrial function was determined by measuring the oxygen consumption rate (OCR) in real time using a Seahorse Extracellular Flux Analyzer XFe96 (Agilent). Cells were seeded in an XF96 microplate at 3 × 10³ cells/well. The culture medium was changed to Seahorse XF DMEM buffer (Agilent) containing 10 mM glucose, 2 mM glutamine, and 1 mM pyruvate, and the plate was incubated at 37°C in a non-CO₂ incubator for 1 h. The compounds from the Seahorse XF Cell Mito Stress Test Kit (Agilent) were loaded into the injection port to reach the final concentrations of 1 μM oligomycin, 2 μM carbonyl cyanide-*p*-trifluoromethoxyphenylhydrazone (FCCP), 0.5 μM antimycin A, and 0.5 μM rotenone to detect respiratory parameters, including basal respiration, ATP production-coupled respiration, maximal respiration, and spare respiratory capacity. The OCR in each microplate well was normalized to the total protein of each well.

The glycolytic function was evaluated by measuring the extracellular acidification rate (ECAR) in real time using a Seahorse Extracellular Flux Analyzer XFe96 (Agilent). Briefly, cells were seeded in an XF96 microplate at 3×10^3^ cells/well. The culture medium was changed to Seahorse XF DMEM buffer (Agilent) and the plate was incubated at 37°C in a non-CO2 incubator for 1 h. The compounds were loaded into the injection port in sequence to reach the final concentration of 10mM glucose, 2μM oligomycin, and 0.1M 2-Deoxy-D-glucose (2-DG). The final ECAR results were normalized to total protein.

#### ROS measurement

The intracellular levels of ROS in cells were assessed by ROS assay kit (Beyotime) that contains DCFH-DA fluorescent probe. Briefly, cells were seeded in 12-well plates and induced into osteoblastogenic differentiation for 14 days. Subsequently, washed cells were incubated with 10 μM DCFH-DA for 20 min in dark and then harvested and washed with PBS. Finally, the intensity of DCFH-DA fluorescence was determined by flow cytometry (BD FACSLyric) at 480 nm (excitation) and 530 nm (emission).

#### Detection of mitochondrial membrane potential

MMP was assessed using Image-iT tetramethylrhodamine (TMRM) reagent (Invitrogen) following the manufacturer’s instructions. In brief, cells were seeded in 35 mm glass-bottom microwell dishes (MatTek) and induced to undergo osteoblastogenic differentiation for 14 days. Subsequently, the washed cells were incubated with 100 nM TMRM reagent and 0.1 mg/mL of the nuclear stain Hoechst 33342 (Thermo Scientific) at 37°C in an incubator for 30 min, followed by washing with PBS. Finally, the microscope was configured to image TMRM (Ex 548/Em 574) and Hoechst 33342 (Ex 350/Em 460), and images were acquired from multiple sites per well at 20× magnification for fluorescence expression using a laser scanning confocal microscope (LSM880, ZEISS).

#### Mitochondrial superoxide measurement

Mitochondrial superoxide in cells was detected by MitoSOX Red mitochondrial superoxide indicator (Invitrogen). Cells were seeded in 35 mm glass bottom microwell dishes (MatTek) and induced to undergo osteoblastogenic differentiation for 14 days. Subsequently, the washed cells were incubated with 5 μM MitoSOX and 0.1 mg/mL of the nuclear stain Hoechst 33342 (Thermo Scientific) at 37°C in an incubator for 10 min, followed by washing with PBS. Finally, the microscope was configured to image TMRM (Ex 510/Em 580) and Hoechst 33342 (Ex 350/Em 460), and images were acquired from multiple sites per well at 20× magnification using a laser confocal microscope (LSM880, ZEISS).

#### Detection of mitophagy *in vitro*

To visualize mitophagy using a mitophagy-specific probe, MCT3E1 cells were transfected with pMT-mKeima-Red (MBL). Keima in neutral conditions was observed using excitation of 430 ± 20 nm and emission of 624 ± 20 nm. Keima in acidic conditions was observed using excitation of 562 ± 20 nm and emission of 624 ± 20 nm. Images were obtained by an LSM880 confocal microscope (Zeiss).

#### Transmission electron microscopy

For primary fixation, MC3T3-E1 cells were enriched by centrifugation, then immersed in 2.5% glutaraldehyde and stored at 4°C for 24 h. The samples were washed with 0.1 M phosphate buffer for 15 min, three times, and then immersed in 1% osmium tetroxide for 2 h for post-fixation. For dehydration, the samples were washed with 0.1 M phosphate buffer for 15 min, three times, and then step-by-step immersed in dehydrating agents (50% ethanol, 75% ethanol, 80% ethanol, 95% ethanol, 100% ethanol, and 100% acetone). The samples were immersed in infiltration agent A (a 2:1 solution of acetone and 812 embedding agent) for 2 h, then in infiltration agent B (a 1:2 solution of acetone and 812 embedding agent) overnight. For embedding, the samples were placed into a mold with embedding medium and then baked at 37°C and 60°C for 12 h, respectively. The samples were cut into sections (60–80 nm thick). For lead-uranium double staining, the sample sections on the copper grid were immersed in a 2% uranyl acetate staining solution for 30 min in the dark, and then in lead citrate staining solution for 15 min. Image Acquisition: The organelle morphology was captured using the JEM-1400Flash electron microscope (JEOL).

#### Bone morphogenetic protein 9 intervention for STZ-induced diabetic osteoporosis in the mouse model

Mice assigned to the STZ group were administered low doses of STZ at 40 mg/kg for five consecutive days to induce diabetic osteoporosis. Mice in the control group were injected with a sodium citrate solution. Mice assigned to the STZ+BMP9 group were administered STZ for five consecutive days, then were administered AAV-BMP9 (Hanbio) at week 5. Specimens were collected at week 18, and elevated BMP9 levels were maintained until the mice were euthanized.

#### Micro-CT analysis and three-point bending test

All procedures involving micro-CT were performed according to the recommendation of the American Society for Bone and Mineral Research.^44^ Briefly, the right femurs of mice were isolated, fixed in 4% paraformaldehyde for 48 h, and then maintained in 75% ethanol. Quantitative analysis of distal femoral metaphysis and midshaft femoral diaphysis were performed using a high-resolution *ex vivo* micro-CT scanner, Skyscan 1176 (Bruker). Using 2D data from scanned slices, 3D analysis was performed to calculate morphometric parameters by a microCT Software (Bruker). The following trabecular morphometric indexes were analyzed: vBMD, percentage of bone volume (BV/TV), trabecular number (Tb.N), trabecular thickness (Tb.Th), trabecular separation (Tb.Sp) and structure model index (SMI). The cortical thickness (Ct.Th) was assessed at the midshaft of femurs.

For biomechanical testing, the left femurs were cleaned of adherent tissue, wrapped in saline-soaked gauze, and tested immediately. A three-point bending test was carried out at the midshaft of the femurs using a mechanical testing machine, the Instron 5569 (USA). The elastic modulus, bending stiffness, maximum bending load, and fracture energy were evaluated.

#### Serum levels of bone turnover markers

Mice were fasted for at least 12 h before being euthanized. Blood was collected and allowed to clot at room temperature for 1 h, then centrifuged at 4,000 rpm for 15 min at 4°C. Serum samples were stored at −80°C. Serum levels of bone turnover markers were determined using enzyme-linked immunosorbent assay (ELISA) kits for mouse PINP (USCN, Wuhan, China), CTX-I (USCN, Wuhan, China), and BMP9 (RayBiotech, USA).

#### H&E staining

The fixed lumbar spines of mice were decalcified in 23% ethylenediaminetetraacetic acid at 4°C for 5–7 days and embedded in paraffin. Bone sections (4 μm) were deparaffinized and washed with water for 3 min. The bone section was stained with hematoxylin staining solution (Beyotime) for 10 min, then washed with water and hydrochloric acid ethanol for 30 s, counterstained with eosin solution, and dehydrated.

#### TSA-enhanced multiplex immunohistochemistry staining

The expression of Drp1 and PINK1 in bone was investigated using multiplex immunohistochemistry staining enhanced by tyramide signal amplification. Bone tissue was prepared into paraffin sections. The bone sections were blocked with 5% BSA and then washed three times with PBS. The sections were incubated overnight in a dilution of anti-Drp1 antibody. After washing three times with PBS, the sections were incubated in a dilution of HRP-linked anti-mouse secondary antibody. Following another round of washing with PBS, the sections were incubated in a dilution of AF 647 tyramide for 10 min. Consistent with the above process, the bone sections were stepwise incubated in a dilution of anti-PINK1 antibody, HRP-linked anti-mouse secondary antibody, and CF 488 tyramide. Finally, the bone sections were incubated in antifade mounting medium with DAPI. The bone sections were scanned using the TissueFAXS system (TissueGnostics, Austria).

### Quantification and statistical analysis

Flow cytometry data were analyzed with FlowJo 10, and fluorescence intensity was quantified by ImageJ software.

Two-tailed Student’s t tests and two-tailed Mann-Whitney non-parametric tests were used for statistical analysis and were performed using GraphPad Prism 9. Specific statistical analyses used are described in the figure legends, and *p*-values are listed in the graphs. All data collection and analyses were conducted in a blinded fashion.
