# Supplementary material for: The inhibition of PINK1/Drp1-mediated mitophagy by hyperglycemia leads to impaired osteoblastogenesis in diabetes
Source: iScience. 2024 Dec 3;28(1):111519. doi: 10.1016/j.isci.2024.111519 (PMC11699391; doi:10.1016/j.isci.2024.111519)
Supplement: Document S1. Tables S1 and S2 [file mmc1.pdf]

iScience, Volume *28*

Supplemental information

The inhibition of PINK1/Drp1-mediated mitophagy by hyperglycemia leads to impaired osteoblastogenesis in diabetes

Xiao-jing Chen, Yu-ying Yang, Zheng-can Pan, Jing-zun Xu, Tao Jiang, Lin-lin Zhang, Ke-cheng Zhu, Deng Zhang, Jia-xi Song, Chun-xiang Sheng, Li-hao Sun, Bei Tao, Jian-min Liu, and Hong-yan Zhao

Table S1: siRNA oligonucleotide sequences, related to STAR methods.

genes	Forward (5 '-3')	Reverse (5 '-3')
mice Pink1-siRNA1	GCAGUUACUUACAGAAGAUTT	AUCUUCUGUAAGUAACUGCTT
mice Pink1-siRNA2	CUGGCUGACUAUCCUGAUATT	UAUCAGGAUAGUCAGCCAGTT
mice Drp1-siRNA1	CCAACAGAAAUGGAACAAATT	UUUGUUCCAUUUCUGUUGGTT
mice Drp1-siRNA2	CAGUCAAGCUUGGAAUAAUTT	AUUAUUCCAAGCUUGACUGTT

Table S2: Primer sequence of target genes in qRT-PCR, related to STAR methods.

species	genes	Forward (5 '-3')	Reverse (5 '-3')
mice	36B4	GAAACTGCTGCCTCACATCCG	GCTGGCACAGTGACCTCACACG
mice	Runx2	CCATGTTGTGGGTGTCTGAG	ACAGGCTGAGTCTGGAGCAT
mice	Osx	GCGACCACTTGAGCAAACATCA	ACCTTCCTCTACCCAGCTCAGA
mice	Ocn	AAGGTAGTGAACAGACTCCGGC	CTCGTCACAAGCAGGGTTAAGC
mice	Alp	CCAGGGGTACAAGGCTAGATGG	AGTTCAGTGCGGTTCCAGACAT
mice	Col1a1	CAAGAATGGCGATCGTGGTGAG	AGACCAGAGAAGCCACGATGAC
mice	Drp1	TCAGATCGTCGTAGTGGGAA	TCTTCTGGTGAAACGTGGAC
mice	NRF1	AGCACGGAGTGACCCAAAC	TGTACGTGGCTACATGGACCT
mice	NRF2	TCTTGGAGTAAGTCGAGAAGTGT	GTTGAAACTGAGCGAAAAAGGC
mice	TFAM	GGAATGTGGAGCGTGCTAAAA	ACAAGACTGATAGACGAGGGG
mice	Pgc1α	CCCTGCCATTGTTAAGACC	TGCTGCTGTTCCTGTTTTC
mice	ND1	TCCGAGCATCTTATCCACGC	GTATGGTGGTACTCCCGCTG
mice	16s	AATTTCGGTTGGGGTGACCT	AGGATTGCGCTGTTATCCCT
mice	Pink1	CACACTGTTCCTCGTTATGAAGA	CTTGAGATCCCGATGGGCAAT
mice	NIX/BNIP3L	ATGTCTCACTTAGTCGAGCCG	CTCATGCTGTGCATCCAGGA
mice	BNIP3	TCCTGGGTAGAACTGCACTTC	GCTGGGCATCCAACAGTATTT
mice	FUNDC1	CCCCCTCCCCAAGACTATGAA	CCACCCATTACAATCTGAGTAGC
